# Enhanced Integrated Gradients: improving interpretability of deep learning models using splicing codes as a case study

**DOI:** 10.1186/s13059-020-02055-7

**Published:** 2020-06-19

**Authors:** Anupama Jha, Joseph K. Aicher, Matthew R. Gazzara, Deependra Singh, Yoseph Barash

**Affiliations:** 1 Department of Computer and Information Science, School of Engineering and Applied Science, University of Pennsylvania, Philadelphia, USA; 2 Department of Genetics, Perelman School of Medicine, University of Pennsylvania, Philadelphia, USA

**Keywords:** Deep learning, Splicing code, Interpretation, Liver-specific splicing, A1CF

## Abstract

Despite the success and fast adaptation of deep learning models in biomedical domains, their lack of interpretability remains an issue. Here, we introduce Enhanced Integrated Gradients (EIG), a method to identify significant features associated with a specific prediction task. Using RNA splicing prediction as well as digit classification as case studies, we demonstrate that EIG improves upon the original Integrated Gradients method and produces sets of informative features. We then apply EIG to identify A1CF as a key regulator of liver-specific alternative splicing, supporting this finding with subsequent analysis of relevant A1CF functional (RNA-seq) and binding data (PAR-CLIP).

## Background

The high accuracy of deep neural networks (DNN) in areas such as computer vision, natural language processing, and robotics has led to the fast adaptation of DNN in biomedical research. In genomics, deep learning models have outperformed previous state-of-the-art methods on tasks such as predicting protein binding sites [1] or mRNA alternative splicing from genomic sequence features [2]. However, the interpretation of these complex models remains a challenge [3, 4]. Approaches to model interpretation include approximation with simpler models [5], identifying the most influential samples [6], or finding the most relevant features for a specific sample or a task by a variety of metrics [7]. Here, we focus on the last approach, which is naturally appealing for biomedical tasks. In this context, interpretability is defined as attributing the prediction of a DNN to its input features. We focus on the recently developed method called Integrated Gradients (IG) [8]. Both IG and DeepLIFT, which was recently used for protein DNA binding sites [9], identify features associated with a model’s prediction for a sample with respect to a baseline. The usage of a baseline is appealing as it serves as the model’s proxy to human counterfactual intuition. This implies that humans assign blame for difference in two entities on attributes that are present in one entity but absent in the other. IG computes feature attribution by aggregating gradients along a linear path between the sample and the baseline. Compared to other interpretation methods, IG offers two desirable theoretical guarantees motivating its usage. The first is *sensitivity*, which states that for every input and baseline that differ in one feature but have different predictions, the method will give a non-zero attribution for that differing feature. The second is *implementation invariance*, which states that regardless of network architecture, if two models are functionally equivalent (same output given any input), then their feature attributions will also be equivalent (see more details in [8]).

While IG has been shown to excel on object recognition problems, it still has several limitations. First, IG only provides feature attributions for individual features with respect to a specific sample. There is no formal mechanism for identifying significant features for a class of interest. Second, IG takes a linear path between the baseline and the sample. The authors speculate that paths visiting points far-removed from actual points seen in training could lead to attribution artifacts. This concern could also happen for linear paths, particularly in high-dimensional datasets where observed data may lie close to a hidden lower-dimensional nonlinear structure in the original feature space (i.e., the manifold hypothesis). In such a case, a nonlinear path taken close to the observed training data might be preferable. Finally, IG attributions are based on features that distinguish a given sample from an all-zero, or no signal, baseline point. This creates two potential issues for the genomics domain. First, in many genomics applications, it is unclear what inputs actually reflect no signal and a zero might not be an appropriate reference point. For example, an exon length of 0 is not biologically meaningful. Second, following the counterfactual argument above, features that distinguish two classes of samples are arguably more useful than a no signal baseline. Such features can elucidate biological clues that make two classes of samples different. For example, features associated with RNA binding proteins that distinguish differentially included cassette exons in the brain from constitutively spliced exons can help shed light on brain-specific RNA splicing regulation.

Here, we systematically address these limitations of IG in a framework we term Enhanced IG (EIG). Specifically, we propose a statistical test to assess significant feature attributions, explore several possible definitions of informed reference points in either the original space or a learned embedding of the samples, and combine these reference points with nonlinear paths to the sample of interest. We first introduce these new additions using models for the handwritten digit recognition task [10] to demonstrate the applicability of our ideas in an easy to visualize domain. We then move to systematically assess EIG using RNA alternative splicing (AS) code models as the main usage case [2].

Predicting RNA splicing outcome from genomic sequence has been the subject of numerous works, originally focused on distinguishing alternative from constitute exons [11]. In the context of splicing codes discussed here, the task is to predict tissue-specific splicing of exons. Specifically, given a triplet of exons in the pre-mRNA, the middle exon (cassette exon) can be either included or skipped in the mature mRNA, giving rise to different isoforms. The splicing code model predicts the middle exon’s inclusion levels (*Ψ*∈[0,1]) and differential inclusion (*ΔΨ*∈[−1,1]) between different conditions (e.g., brain vs. liver) as a function of a 1357-dimensional feature vector (see Additional file 1: Table S1-S3 and Additional file 1: Fig. S1 for details about the architecture of this splicing code model). These features are parsed from genomic regions containing the exon triplet and flanking introns. They include sequence conservation scores, nucleosome positioning, *k*-mer frequencies, splice site strength, and *cis*-acting regulatory motifs. Cis-acting regulatory motifs can serve to recruit *trans*-acting RNA binding proteins (RBPs) to promote or inhibit inclusion of an exon in a transcript. RBPs are known to regulate alternative splicing through direct binding to their target or indirect regulation through other RBPs. For example, Rbfox and Nova are RBPs that have been shown to bind proximal to alternative exons and promote their inclusion or exclusion in adult brain and in neurogenesis [12]. Nova has been shown to bind clusters of short YCAY motifs [13] while Rbfox binding site is commonly defined by the [U]GCAUG consensus motif. However, Rbfox has also been shown to operate as part of the LASR complex without requiring the above binding motif [14], and recent work also points to secondary motifs by which Rbfox may exert its function[15], illustrating some of the complexities involved in deciphering a predictive splicing code in silico.

The splicing quantification (*Ψ*,*ΔΨ*) used here to train and test the models were derived from RNA-seq experiments involving six mouse tissues (hippocampus, heart, liver, lung, spleen, and thymus) [16] and quantified using MAJIQ [17]. Specifically, for the results described below, we use a group of cassette exons that are differentially included in the hippocampus as the set of interest. These alternative exons should be enriched in features informative for differential inclusion in the hippocampus. We select constitutively spliced exons that are always included in all tissues as the baseline class. Constitutive exons are chosen as the baseline since these events differ from alternative events in both core splicing features and brain-specific splicing features.

Models for the splicing prediction task described above have several desirable characteristics for analyzing DNN interpretation. First, the features are an identifiable set representing prior biological knowledge about putative regulatory elements such as known sequence motifs and RNA conservation scores. Moreover, a myriad of models have already been applied to this task, including a mixture of decision trees, Bayesian neural networks, naive Bayes, and logistic regression [18, 19]. Second, the splicing code model includes embedding in a lower dimension space, a common component in genomic models, allowing us to test the usage of feature embedding for prediction attribution.

Using splicing codes as a test case, we assess different configurations of EIG, simple gradients, and IG. We also include SHAP, a recent method that offers a generalization over several previous interpretation methods using the framework of Shapley values [20]. Specifically, DeepLIFT and SHAP have been combined into Deep SHAP, an approximate algorithm for computing SHAP values for deep learning models. For these methods, we assess the number of significant features and their relative enrichment in known regulatory features. We then assess the effect of the number of selected features on prediction accuracy.

Finally, we use EIG to study liver-specific splicing regulation. EIG identifies A1CF as a possible liver-specific splicing factor. We employ several downstream analyses to support this finding. These include splicing quantification from a recent *A1cf* knockout mouse model, motif maps showing positional bias and enrichment of the A1CF binding motif around liver regulated exons, and PAR-CLIP peak mapping to support direct liver-specific regulation by A1CF.

## Results

### Nonlinear paths, meaningful baselines, and feature significance framework improve interpretation of digit recognition

**Fig. 1:**
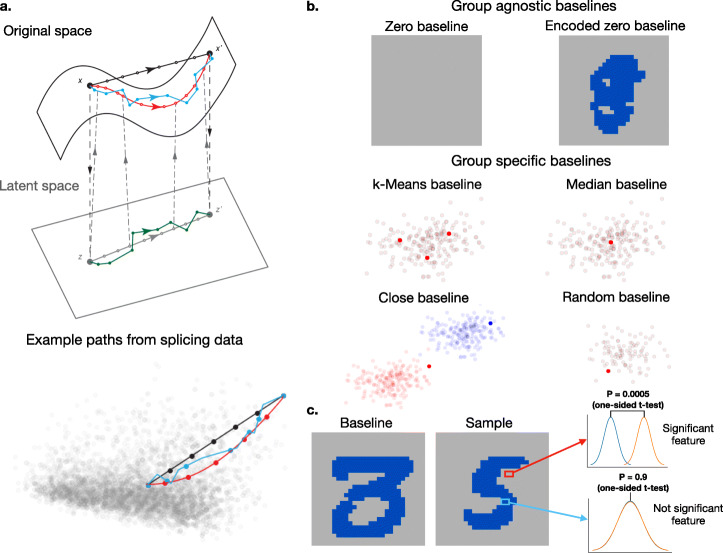
EIG enhancements to identify relevant features per sample or task. **a** Top: Different linear and nonlinear paths for EIG. (1) Linear path in original feature space (O-L-IG, black line), (2) neighbors path in the original feature space (O-N-IG, blue line), (3) linear path in the hidden feature space (H-L-IG, gray line), and (4) neighbors path in the hidden feature space (H-N-IG, green line). Bottom: visualization of such paths for a specific sample using splicing data from [16] and PC1, PC2 of the feature space (see Additional file 1: Section S1). **b** Different group-agnostic (zero and encoded-zero) and group-specific (*k*-means, median, close, random) baselines. Encoded-zero baseline is generated by decoding a zero vector in the latent space. A close baseline is created by taking a baseline point which is close to the sample in euclidean distance (see the “Methods” section). **c** Framework to identify significant features that distinguish sample 5 from baseline 3. The digit images show mean of 300 examples of sample digit 5 and median of 300 examples of baseline digit 3. The distribution plots are illustrative only. They show difference between distribution of attributions of two sets of samples for a significant and a not significant feature

IG is based on approximating the integral over the gradient, per feature, between two points: the sample of interest and a reference point. In the original IG formulation by [8], only linear paths between these two points were used. In this work, we evaluate several alternative path formulations. As seen in Fig. 1a, these paths are as follows: (1) linear path in original feature space (O-L-IG, black line) as in the original IG, (2) *k*-nearest neighbors path in the original feature space (O-N-IG, blue line), (3) linear path in the hidden feature space (H-L-IG, gray line), and (4) *k*-nearest neighbors path in the hidden feature space (H-N-IG, green line). Here, hidden feature space refers to the encoded representation produced by encoder of an autoencoder model. For H-L-IG and H-N-IG paths, a trained autoencoder is required for encoding features from original to hidden feature space and decoding features from hidden to original feature space. Using the latent embedding from the trained autoencoder as input, we train the downstream supervised learning task (handwritten digit recognition and splicing prediction). Then, we combine the encoder from the autoencoder along with the downstream network. The combined encoder and downstream network architecture enables us to design paths in the original and hidden space.

We also assess several approaches to define a reference point, or groups of those, for the different linear and nonlinear paths. First, we consider a *group-agnostic* reference which does not require any prior biological information to define it. Specifically, an encoded-zero baseline (encoded-zero O-L-IG, Fig. 1b top panel) is generated by decoding the zero vector in the hidden space of our autoencoder. We also evaluate several approaches to define a *group-specific* baseline using different methods for selecting reference points (*k*-means, median, close, and random) as shown in Fig. 1b, bottom panel. In principle, these baseline points can be chosen either in the original or hidden feature space, but here, we chose to define these baseline points in the original feature space.

Finally, we include a significance test procedure to identify significant features associated with a prediction task. This procedure first computes the relative ranking of a feature’s attribution across samples belonging to a class of interest. Then, these rankings for a similarly sized random set of samples are computed. The two sets of relative ranking are then compared using a one-sided *t* test with Bonferroni correction for multiple testing (an illustration is shown in Fig. 1c) to identify the set of significant features.

**Fig. 2:**
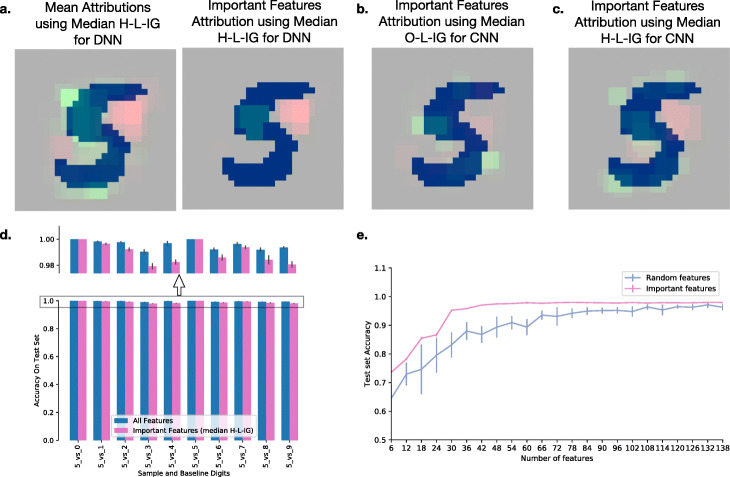
EIG framework with handwritten digit data. **a** On the left, mean attributions generated from 300 examples of digit 5 and median baseline digit 3 using median H-L-IG approach on a feed-forward neural network. On the right, the subset of statistically significant features for the same set (one-sided *t* test, Bonferroni adjusted *p* value ≤ 0.05.) Pixels belonging to the digit 5 are blue, positive attribution shown in green, and negative attribution shown in red. **b** Statistically significant features for distinguishing digit 5 from baseline 3 using median O-L-IG approach on a convolutional neural network (CNN). For generating the attributions, linear path was computed in the original feature space (O-L-IG) from a median baseline. **c** Statistically significant features for distinguishing digit 5 from baseline 3 using median H-L-IG approach on a CNN with a convolutional variational autoencoder (C-VAE). For generating the attributions, linear path was computed in the latent space (H-L-IG) using the C-VAE. **d** Performance of models trained to distinguish sample from the baseline digits using all features or only the significant features identified using our approach (0.00 to 1.44% loss in accuracy while using 8 to 20% of all pixels). The top panel enlarges the *y*-axis (0.98 to 1.00) to highlight the differences in performance. These models solve the binary classification task of distinguishing the sample digit from the baseline digit and thus require fewer pixels than the original multi-class classification problem of classifying each image in as one of ten possible digits. **e** Test set accuracy of models trained to distinguish digit 5 from baseline 3 with increasing subsets of significant features (pink) or random features (blue). *x*-axis shows increasing subsets of features, and *y*-axis shows the accuracy on the test set

We illustrate the usefulness of our prediction attribution framework using the visually intuitive task of handwritten digit recognition [10]. Using the MNIST dataset, we first create a joint model from a variational autoencoder and a feed-forward network. The digit model predicts the identity of a handwritten digit between 0 and 9 from a 28×28 pixel image (see Additional file 1: Table S4-S6 and Additional file 1: Fig. S2 for details about the architecture). Using this network, we generate attributions for the sample digit 5 from the baseline digit 3. Figure 2a left panel shows mean attributions across 300 examples of 5 from median baseline 3 using the linear path in the hidden space. We can see that to distinguish the digit 5 from the baseline 3, median H-L-IG identifies that the pixels on the bottom left and the top right should be absent (red) while the top left should be present (green). When combined with the significant feature selection procedure, these results become more pronounced, while other weaker attributions are removed (Fig. 2a, right panel).

While we make use of a variational autoencoder to generate embeddings for H-L-IG paths in the example described, other components of EIG, e.g., baselines, linear, and neighbors path, in the original feature space (O-L-IG and O-N-IG) and the significance framework can be run on any deep neural network without need of an autoencoder. To show this along with application of EIG to convolutional neural networks (CNNs), we train two convolutional neural networks on the task of handwritten digit recognition. For both of these, since the CNN scans patches (3 ×3 pixels) in the image, we assess significant patches rather than individual pixels. First, Fig. 2b shows the significant features for distinguishing the sample digit 5 from the baseline digit 3 using a CNN with no additional requirement of an autoencoder. The computed paths here are from a median baseline using linear paths in the original feature space (O-L-IG). Similar to the result from Fig. 2a, right panel, we observe pixels that should be absent (red) and present (green) in regions of a digit 5, though these seem to stand out less. Next, we train a convolutional variational autoencoder (CVAE) to reconstruct the handwritten digit images. Then, using the latent embedding from this CVAE as the first layer, we train a feed-forward network for handwritten digit prediction. Finally, we combine the encoder from CVAE and the feed-forward network to produce the final network. For this task, we produced significant features using again a median baseline but the linear path is in the hidden space. Comparing the result for this network (Fig. 2c) to those in Fig. 2b, we can see that the pixels that should be absent (red) are arguably better captured by this combined CVAE and feed-forward network but neither of those appear better than the results for a DNN with H-L-IG (Fig. 2a).

### Identified significant features have better predictive power for handwritten digit recognition

More generally, to test whether our framework identifies significant features that can distinguish the sample class from the baseline class, we train networks using only significant pixels (8 to 20% of all pixels) as features on the task of distinguishing the sample digit 5 with each remaining digit serving as baseline in a different network. Figure 2d shows that the accuracy of this model on the test set is almost as good (0.0 to 1.44% loss in accuracy) as a network trained with all 784 pixels as features.

Finally, we wish to evaluate the predictive power of significant features found using EIG (significant features generated with median baseline 3, sample digit 5 using linear path in latent space on the feed-forward network with variational autoencoder). Therefore, in Fig. 2e, we plot the predictive performance of a feed-forward network on the binary classification task for digits 5 and 3 with increasing subset of significant pixels (pink) or random pixels (blue). As can be expected, the pink curve using significant pixels has higher accuracy and saturates earlier than the blue curve using random pixels, indicating that the identified significant pixels have better predictive power.

### Establishing a robust framework for identifying significant splicing code features

As described in the introduction, training the splicing code models involves the training of an autoencoder or variational autoencoder as the first step. Thus, any downstream analysis should first address the stability of these nonlinear embeddings in a lower-dimensional space. Given our interest in paths between points and the advantages demonstrated in other domains for assessing similarities based on local structures [21–23], we test stability of the embeddings via neighborhood similarity. Specifically, we evaluate several autoencoder and variational autoencoder architectures to conclude the embeddings do not vary much, with an observed Spearman rank correlation typically in the range of 0.70–0.95 when varying architectures, bootstrapping samples, and initializations (see the “Methods” section and Additional file 1: Fig. S3).

Using the latent embedding from the trained autoencoder as input, we train a feed-forward network with similar architecture as [2] (see Additional file 1: Fig. S1 for details about the architecture). The combined encoder and feed-forward network is then used to predict the splicing outcome. This combined architecture enables us to run attribution in the original and hidden space. We also address a potential stability issue of the integral approximation performed by IG. Based on this analysis, we select 250 points for the experiments described below (see Additional file 1: Section S2 and Additional file 1: Fig. S4 for details on path interpolation).

To identify significant features for any group of splicing samples, we first group highly correlated features (e.g., different versions of the Rbfox splicing factor binding motif) into *meta-features* in order to avoid spreading attributions associated with the same entity (Rbfox binding in this example). This results in the 1357 splicing features grouped into 781 meta-features. The significance test procedure described above is then applied at the level of these meta-features to the sum of associated features attribution to avoid inflating the significance of large meta-features.

### Monitoring *p* value distribution for EIG feature attribution, combined with prediction accuracy curves, offers insights on the informative features set

To ensure that our *p* values are well calibrated, we compared two similar sized random group of events. It produced well-calibrated *p* values with no feature passing a *p* value of 0.05 after multiple hypothesis correction (Additional file 1: Fig. S5). It is instructive to compare this distribution to the *p* value empirical distribution on real data. In the case of the digit recognition task (Additional file 1: Fig. S6, left), the empirical *p* value distribution is clearly skewed by a small set of highly informative features (*p*∼0) and a set of “dead” pixels (*p*∼0.5). Removing those along with the dead pixels set results in a much closer to uniform distribution (Additional file 1: Fig. S6, right).

Another observation to be made here is that although the identified significant pixels are only a small fraction of the total number of features, the early saturation of the pink curve in Fig. 2e is likely to be the result of the high correlation between many of the pixels. This correlation, and subsequent redundancy in terms of predictive capacity, can be gleaned from the regions of green and red patches in Fig. 2a–c. Thus, while the identified pixels may indeed be informative for the task at hand and represent a sparse solution in feature space, they are not intended or optimized to provide a minimal feature set, as is the focus of some other recent work [24].

In summary, we found that monitoring that *p* value distribution for EIG feature attribution, combined with prediction accuracy curves, can offer insights on the informative features set detected by EIG. We next turn to focus on the splicing code models as a case study.

### EIG with nonlinear paths identify significant features missed using linear paths or simple gradients

**Fig. 3:**
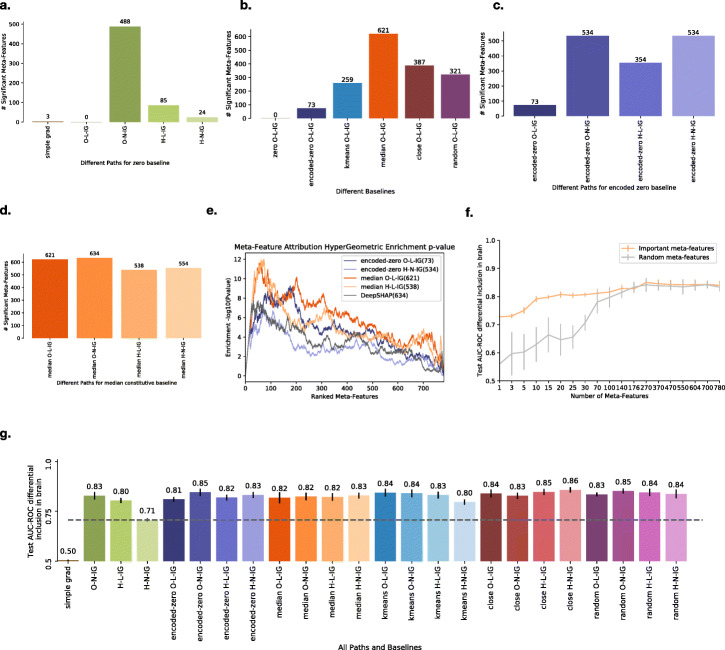
Performance evaluation of IG, EIG, and Deep SHAP on splicing data. **a** Number of significant meta-features identified by different methods: simple gradients, original IG (O-L-IG), and EIG nonlinear paths (see main text) with a zero baseline. **b** Number of significant meta-features identified by different EIG baselines (explained in text) with a linear path in the original feature space. **c** Number of significant meta-features identified by different EIG paths with an encoded-zero baseline. **d** Number of significant meta-features identified by different EIG paths with three median-constitutive baseline points. **e** Enrichment of known brain regulatory features in significant features identified by the best two EIG paths each from encoded-zero and median-constitutive baselines and Deep SHAP. **f** Splicing prediction for differential inclusion in the brain with increasing subsets of significant meta-features identified by EIG with latent-linear path (H-L-IG) and median baseline from constitutive splicing events (orange line) or random meta-features (gray line). The *x*-axis shows the number of meta-features. The *y*-axis shows the AUC-ROC for differential inclusion on the test set. Significant features passing one-sided *t* test, Bonferroni adjusted *p* value ≤ 0.05. **g** Splicing prediction for differential inclusion in the brain using features found to be significantly generated by different combinations of paths (O-L-IG, O-N-IG, H-L-IG, H-N-IG) and baselines (zero, encoded-zero, median, k-means, close, random) and simple gradients. *x*-axis shows the different interpretation methods, and *y*-axis shows the AUC-ROC for differential inclusion in the brain on the test set

Using our significance framework, we first evaluate the effect of different nonlinear paths. Figure 3a shows that simple gradients and the original IG as proposed in [8], linear path in original feature space with zero baseline (O-L-IG), fail to identify any known regulatory features as significant. In contrast, nonlinear paths, O-N-IG, H-L-IG, and H-N-IG, identify 488, 85, and 24 meta-features as significant, respectively.

It is admittedly hard to assess levels of false-positive features for such a real-life prediction task or to argue that identifying more features (e.g., 488 vs. 24 as above) is strictly better. Nonetheless, the lack of significant features when using simple gradients or IG is striking. Furthermore, previous modeling efforts reported hundreds of those features as relevant and these features include many known regulatory features such as conservation scores and known binding motifs of brain-specific RNA binding proteins that we expect to find [18].

### Group-agnostic and group-specific baselines identify significant features missed by a zero baseline

Next, we evaluate several approaches to define a reference point, or groups of those, for the different linear and nonlinear paths. For group-agnostic reference, encoded-zero baseline identifies 73 significant meta-features, a marked improvement over the original zero O-L-IG which failed to identify any brain-specific features. When the encoded-zero baseline is combined with nonlinear paths, a higher number of significant features are identified (354–534) as shown in Fig. 3c.

The group-specific baselines for the splicing prediction task described here are designed to find significant features that distinguish differential inclusion in the brain from constitutive splicing events. This means that the identified features should make a specific splicing event both an alternative exon (e.g., weak splice sites) and differentially included in the brain (e.g., Nova motif clusters). This refined definition of the baseline results in a larger number of significant meta-features being identified compared to those found by the group-agnostic baseline, with median O-L-IG identifying the highest number of significant meta-features (621, Fig. 3b). Notably, we found that median baseline finds a similar number of significant features across all linear and nonlinear paths (Fig. 3d, see Additional file 1: Fig. S7 to see performance of other baselines). In addition, when we tested for the overlap of the features reported as significant by the various path and reference point definitions, we found extensive overlap indicating robustness (Additional file 1: Fig. S8). For example, models that used a group-agnostic baseline of encoded-zero only had 4/534 shared features which were not reported by the models using a group-specific baseline.

While we tested for identified feature *p* value calibration in order to control for false positives (Additional file 1: Fig. S9), reporting more informative features is not necessarily an indication of improved quality of a method. Since different EIG paths and baselines produce different number of significant features, we need to assess the quality of these features. Therefore, we perform two evaluations to test the quality of features found by EIG paths and baselines: enrichment of known biological features in the set of found features and the predictive performance using the chosen significant features. We describe each of these criteria in the next two sections.

### EIG shows higher enrichment of known biological features than deep SHAP

To get a quantitative assessment of the biological relevance of the reported features, we tested them for enrichment of known splicing features and brain-specific regulatory features that are different from constitutive splicing events (see the “Methods” section). To assess such enrichment, we computed a hypergeometric *p* value for the overlap between this pre-defined feature set and the any subset of each method’s reported features, ordered by their relative attribution. Figure 3e shows that significant meta-features identified by constitutive baselines with linear (median O-L-IG) and nonlinear (median H-L-IG) paths have higher enrichment of these known regulatory features than Deep SHAP. On the other hand, the *group-agnostic* baseline (encoded-zero O-L-IG and encoded-zero H-N-IG) has a similar level of enrichment for these features as Deep SHAP (performance of other EIG methods is summarized in Additional file 1: Fig. S10). We conclude that when compared to the recent Deep SHAP, EIG achieves higher or similar level of enrichment of known regulatory features while reporting similar or fewer features overall.

### Identified significant features improve splicing prediction

As a second criteria to evaluate the quality of reported meta-features, we evaluated their contribution to accurately predicting splicing changes (*ΔΨ*) on the set of exons which are differentially spliced in the brain. These significant meta-features were generated using median baseline constitutive splicing events, against samples from differentially included events in the brain using linear path in latent space on the feed-forward network with an autoencoder (Fig. 3d, third bar). Therefore, in Fig. 3f, we plot the predictive performance of a feed-forward network on this splicing prediction task with increasing subset of significant meta-features (orange) or random meta-features (gray). We find that the orange curve using significant meta-features has higher predictive power and saturates earlier than the gray curve using random meta-features, indicating that the identified significant meta-features have better predictive power. The prediction improvement for the differentially included exons in the test set saturates around 70–100 meta-features. This early saturation has been reported in previous works [18] and is to be expected for several reasons. First, the splicing code feature set is based on putative regulatory features collected from the literature. As such, it is highly enriched for informative features (see also Additional file 1: Fig. S10). Second, there is built-in redundancy between the meta-features in terms of predictive capacity, as discussed above regarding the saturation observed in Fig. 2c for the digit task. For example, high upstream intron conservation is likely to be accompanied by high conservation of the downstream intron. In addition, the selected splicing code features carry information relevant to additional tasks such as identifying alternative exons and distinguishing low-inclusion and high-inclusion exons that are not needed for this task. Overall, this result indicates that 70–100 meta-features are sufficient for peak predictive performance on this task. We notice the same trend when we plot the predictive performance of all EIG methods (paths: O-L-IG, H-L-IG, O-N-IG, and H-N-IG, and baselines: zero, encoded-zero, median, *k*-means, close, random) on the same splicing task of differential inclusion of splicing events in the brain. We find that all flavors of EIG that find more than 70–100 meta-features show similar predictive performance (Fig. 3g). Exceptions are simple gradients (3 meta-features), zero baseline with O-L-IG (0 meta-features), and H-N-IG (24 meta-features) paths.

### Identification of A1CF as a regulator of a liver splicing program

Finally, we wished to use EIG to uncover biological insight into tissue-specific splicing regulation. Early studies noted that certain tissues, like the brain and the liver, exhibited extensive alternative splicing, relative to other tissues [25]. While the brain has since been well studied for regulators of neural splicing patterns (reviewed in [26]), the liver has relatively few characterized splicing regulators to date (reviewed in [27]) with none having liver-restricted expression.

**Fig. 4:**
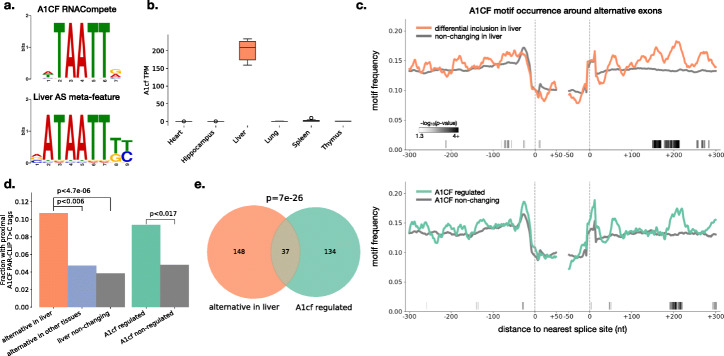
Identification of A1CF as a regulator of the liver splicing program. **a** Tomtom [28] alignment of PSSMs for the in vitro determined binding site of the RNA binding protein A1CF ([29] top) and a significant meta-feature identified in multiple liver versus other tissue comparisons (bottom). **b** Boxplots showing the expression level of *A1cf* in transcripts per million (TPM) in the indicated tissues across the six mouse replicates. **c** Motif maps showing the frequency of 3-mers known to bind A1CF (AAU, UAA, or AUU [29, 34]) around the 3’ and 5’ splice sites of cassette exon sets indicated in the legends. Frequencies were smoothed using a running mean of 20 nucleotides (nts). Grayscale boxes indicate significant differences in motif occurrence (− log10(*p*)) between the regulated versus non-regulated exon sets (*p*<0.05, Fisher’s exact test assessed at sliding windows of 20 nts). **d** Bar chart showing the fraction of exons in the indicated sets that contained evidence of A1CF binding proximal to the cassette exon (within 300 nucleotides upstream, within the cassette exon, and/or within 300 nucleotides downstream). Significance was assessed using a two-tailed Fisher’s exact test. **e** Venn diagram showing the overlap of cassette exons that were alternatively spliced in the liver versus other tissues and those regulated by A1CF, given they were quantified in both experiments. Significance was assessed using a two-tailed Fisher’s exact test

To identify putative regulators of the liver splicing program, we applied EIG to sets of exons that were differentially spliced in the liver versus the other five tissues. Our analysis for enrichment of known biological features showed highest enrichment for median baseline followed by encoded-zero baseline. Therefore, we ran interpretation for the differential inclusion/exclusion in the liver using encoded-zero and median baselines for all four paths (O-L-IG, O-N-IG, H-L-IG, and H-N-IG). We then used Tomtom [28] to align the PSSM of a significant meta-feature identified through this procedure with the in vitro determined motifs of a compendium of RNA binding proteins from RNACompete [29]. This analysis found a significant match to a motif recognized by APOBEC1 complementation factor (A1CF) (Fig. 4a). A1CF is most well characterized as an RNA binding partner of APOBEC1 that contributes to C-to-U RNA editing levels in specific tissues [30]. However, A1CF is part of the hnRNP family of RNA binding proteins that play roles in various aspects of RNA processing [31]. We therefore hypothesized A1CF may have a role in a liver splicing regulatory program, resulting in the associated features identified by our EIG framework. In support of a role for A1CF in splicing regulation, a study published while this manuscript was being prepared reported splicing changes for approximately 80 genes in the liver of a *A1cf* knockout mouse model [32]. We then used the datasets produced in this study to further assess the hypothesis of an A1CF liver-specific splicing program suggested by our initial EIG analysis.

In line with known expression patterns, we observed liver-restricted expression of *A1cf* in the mouse tissues analyzed here (Fig. 4b). Because splicing regulators typically act in position-specific manners to alter spliceosome assembly in regions proximal to alternative exons [33], we next wished to examine the A/U-rich motif frequency (the known binding site for A1CF [29, 34]) around different sets of regulated exons. We compared exons that were differentially included or excluded in the liver to alternative exons for which inclusion does not change in the liver. This comparison identified regions of enriched A1CF motif occurrence proximal to the alternative exon (Fig. 4c top panel, grayscale boxes indicate regions of significant differences based on Fisher’s exact test, see the “Methods” section for details). To focus only on the cassette exons which had differential inclusion between tissues, we also examined A1CF motif occurrence around the set of exons that were differentially included between other tissues but not in the liver, and still observed regions of enriched motif occurrence in the liver-specific set (Additional file 1: Fig. S11a, compare orange and blue). This suggests A1CF binding is not common to all alternative exons, but specifically enriched around exons regulated in the liver.

To see if this liver-specific motif enrichment was consistent with A1CF splicing regulation, we analyzed RNA-seq data from the livers of wild-type and *A1cf* knockout mice [32]. We defined the exons which were regulated and non-responsive to A1CF and plotted motif occurrence around these exons. Although little was previously known about how proximal A1CF binding influences splicing outcomes, we were able to detect regions of enriched A1CF motif occurrence around the A1CF-regulated versus non-regulated exons (Fig. 4c, bottom). Most notably, this included a region around 200 nucleotides downstream of alternative exons that was also enriched in the set deferentially included in the liver (Fig. 4c bottom, compare orange and cyan with grayscale boxes at the bottom indicating statistical significance of motif enrichment). Next, we stratified these exon sets into those with high inclusion in the liver and compared them to exons enhanced by A1CF or exons excluded in the liver and compared them to exons repressed by A1CF, with the expectation that these sets should exhibit similar motif enrichment patterns. While subsetting these exons sets introduced some additional noise, there were still matching regions of enriched motif occurrence around 200 nucleotides downstream of the regulated exons (Additional file 1: Fig. S11b,c). The lack of strong position-dependent effects of the A1CF motifs around the exons it enhances versus represses (e.g., upstream vs. downstream binding) may suggest additional contextual requirements in determining splicing outcomes for A1CF target exons (e.g., additional binding partners, altered or extended binding motif requirements in vivo, splice site strength), and further study is necessary in this regard.

While the above analysis is suggestive, motif occurrence does not guarantee RNA binding and the in vitro determined motifs identified for A1CF may not reflect binding motifs in vivo. To address these concerns, we analyzed A1CF PAR-CLIP data from the mouse liver [32] using the CLIP Tool Kit [35]. We focused our analysis on positions with multiple unique tags containing T-to-C cDNA transitions which are indicative of nucleotide-resolution RNA binding [36]. We saw local enrichment of the in vitro determined A1CF motifs at these cross-linked sites (Additional file 1: Fig. S12a), suggesting A1CF can bind these same motifs in vivo. Moreover, we saw a higher average number unique T >C PAR-CLIP tags at positions consistent with the motif maps for the liver and A1CF exon sets, most strikingly in the region around 200 nucleotides downstream of the regulated exons (Additional file 1: Fig. S12b, Fig. 4c). Additionally, A1CF bound proximally to a significant fraction of both liver- and A1CF-regulated exons compared to relevant non-changing and differentially included in other tissue sets (Fig. 4d). As with the motif analysis, we subsetted regulated exon sets into liver-specific increased or decreased inclusion and compared those to A1CF enhanced and repressed exons to examine position-specific binding effects. While all four regulated exon sets had enriched A1CF PAR-CLIP binding downstream alternative exon when compared to non-responsive exons, this enrichment was more striking downstream of the liver included and A1CF enhanced exon sets, similar to the motif analysis (Additional file 1: Fig. S12c, red). A1CF repressed exons, on the other hand, were also enriched for intronic binding upstream of the alternative exon to a similar degree as the liver repressed exons (Additional file 1: Fig. S12c, blue). However, we note that this upstream intronic binding was also observed in the liver enhanced exons. Finally, we found a significant overlap in the exons regulated by A1CF and the liver alternative exon set (Fig. 4e).

In summary, these results suggest that some additional features may dictate whether A1CF enhances or represses particular exons in the liver. Nonetheless, all lines of enquiry we employed based on motif enrichment, A1CF knockdown, and CLIP data suggest our models correctly identified A1CF as a key, direct regulator of the liver alternative splicing program.

## Discussion

While the usage of deep learning in genomics has grown exponentially in the recent years [37], relatively little work in the genomics literature has addressed the need for interpretation of these models. Here, we define model interpretation in terms of feature attribution, i.e., finding features that are significant for a specific prediction task. We present a framework to identify such features, building upon the method of Integrated Gradients (IG) [8]. Our specific computational contributions are that we propose nonlinear paths, group-agnostic and group-specific baselines, and a statistical test to assess significance of different features. We show these additions improve the feature detection ability of IG for handwritten digit recognition and splicing prediction problems. Additionally, we ensure robustness of our results by assessing stability of data points in latent spaces of different model architectures via neighbor similarity.

The lack of ground truth for many biological datasets is likely a major reason for the scarcity of published work on model interpretability in genomics. Simulated data or image recognition data, where the ground truth is readily available, is often used to get around this issue. For example, to assess DeepLIFT, the authors used MNIST digit recognition data and simulated motif sequences [9]. However, such synthetic data are not necessarily representative of a real-life task and particularly for splicing codes for which simulation procedures are not obvious. Instead, we sidestepped this issue by focusing on a real-life prediction task which involves identifiable features. Admittedly, the evaluation of significant features for this real-life task does not offer direct assessment of false-positive and false-negative features. In addition, our approach does not offer any theoretical guarantees beyond those of sensitivity and implementation invariance discussed before. Specifically, the usage of a reference sample has been shown to overcome issues of activation saturation [9], but we lack theoretical guarantees for identifying significant features when they are highly correlated. Instead, we group highly correlated features (e.g., variants of a splice factor binding motif) and compute the total absolute attribution assigned to all features in that group (see the “Methods” section). More advanced approaches may be applied to handle this issue, but in practice, we found that even comparing the contribution of those redundant features to the null distribution allowed us to identify them as individually significant, indicating robustness.

Despite the limitations discussed above, our approach gave robust and intuitive results on the task of handwritten digit recognition. Furthermore, we were able to demonstrate that our approach controls for false-positive features when compared to a randomly selected reference group, that the identified features give high prediction accuracy when used alone, and that by combining prediction accuracy curves (Figs. 2e and 3f) with empirical *p* value distribution (Additional file 1: Fig. S6,S9), we can learn about both sparsity and redundancy of the informative feature set. Importantly, we showed that the enhancements we propose to IG enable it to identify many more biologically relevant features. Specifically, we find simple gradient and IG with zero baseline perform poorly. Instead, users in search of a group-agnostic baseline can use an encoded-zero baseline. Of the nonlinear paths we offer, H-L-IG is the most robust across different baselines while being computationally more efficient than the O-N-IG and H-N-IG paths. However, connectivity in the latent space, as is observed in single cell data, may motivate neighbor-based approaches in other applications. The performance of baseline selection methods for the group-specific baseline depends on the underlying structure of the data, but using the median points is computationally efficient and yields best results on the splicing data. Finally, when compared to Deep SHAP, we find our approach finds higher enrichment of biologically relevant features.

As a future direction for research, we believe that elaborate and detailed mapping of where and why various interpretation methods succeed or fail will be highly valuable to the machine learning community in general and researchers focused on biomedical tasks in particular. For example, here, we demonstrate that IG did not perform well for the splicing code prediction task, but in another recent work involving protein DNA binding site predictions, IG and mutagenesis analysis performed similarly well [38]. Understanding the effect of the type of features (e.g., binary vs. discrete vs. continuous), how the feature values are distributed, redundancy between features, or other characteristics of the domain could be highly valuable for researchers to discern which model interpretation method they should use for their specific task.

While the above discussion is informative with respect to EIG as an interpretation method, arguably, the ultimate utility of such a method is in its ability to generate high confidence hypotheses for follow-up. Here, we were able to apply EIG to identify A1CF as a regulator of the liver splicing program, then support this result using several lines of experimental and computational evidence based on motif maps, RNA-seq from a *A1cf* knockout in mouse, and PAR-CLIP binding assays.

Overall, this work offers a framework for researchers in genomics to interpret deep learning models and propose specific hypotheses for downstream validation as in the case of A1CF presented here. We hope this framework, along with the available code, will help gain new insights from the deluge of deep models being developed for biomedical tasks.

## Methods

### Notation

We are interested in interpreting the prediction made by a deep learning model given an input observation by assigning attributions to each feature of the observation. Here, we assume that predictions are made from inputs ***x*** in a *p*-dimensional feature space $\mathcal {X}=\mathbb {R}^{p}$. Predictions are obtained by a real-valued prediction function on feature space $F:\mathcal {X}\rightarrow \mathbb {R}$. The goal is to obtain a *p*-dimensional vector of attributions called $\boldsymbol {\text {attr}}\in \mathbb {R}^{p}$, with each coordinate corresponding to the *p* dimensions of the feature space, for an input ***x*** that indicates how each of the *p* features contributes to the prediction *F*(***x***).

### Datasets

In this work, we use RNA-seq experiments processed by [16] from six mouse tissues (hippocampus, heart, liver, lung, spleen, thymus) with average read coverage of 60 million reads. We generated 1357 genomic features from 14,596 exon skipping events and 74,156 constitutive exon triplets using AVISPA [39]. *Ψ* and *ΔΨ* quantification for the exon skipping events were generated using MAJIQ [17]. In addition, we use the MNIST handwritten digit dataset [10]. This dataset contains 70,000 images of handwritten digits from 0 to 9. Each image contains 28×28 pixels.

### Splicing code model

We first train an autoencoder to find a latent embedding for the 1357 splicing features in a lower-dimensional space. Subsequently, we use this embedding along with a separate input for two tissue types as the input to a feed-forward neural network. Predictions are made for three targets:

$$\begin{array}{@{}rcl@{}} T_{\Psi_{e,c}} &=& E[\Psi_{e,c}] \\ T_{\Delta\Psi_{inc,e,c,c^{\prime}}} &=& \lvert \max(\epsilon, E[\Delta\Psi_{e,c,c^{\prime}}])\rvert \\ T_{\Delta\Psi_{exc,e,c,c^{\prime}}} &=& \lvert \min(\epsilon, E[\Delta\Psi_{e,c,c^{\prime}}])\rvert \end{array} $$

T$_{\Psi _{e,c}}$ is the expected PSI value of the event *e* in condition *c*, T$_{\Delta \Psi _{inc,e,c,c^{\prime }}}$ captures the *ΔΨ* for event *e* if it has increased inclusion between condition *c* and *c*^′^, and T$_{\Delta \Psi _{exc,e,c,c^{\prime }}}$ captures the *ΔΨ* for event *e* if it has increased exclusion between condition *c* and *c*^′^. *ε* is a uniform random variable with values between 0.01 and 0.03. It is used to provide small *ΔΨ* values for non-changing events. Our goal is to interpret predictions made by this model, attributing which of the 1357 features contribute to $T_{\Psi _{e,c}}$, $T_{\Delta \Psi _{inc,e,c,c^{\prime }}}$, or $T_{\Delta \Psi _{exc,e,c,c^{\prime }}}$ for a given sequence. Thus, we combine the encoder from the autoencoder and the splicing code feed-forward network to form a single network. The attributions are computed on this combined network.

### Handwritten digit model

We use a feed-forward neural network architecture for this task with a variational autoencoder for dimensionality reduction. The input to the feed-forward neural network is the latent embedding from the variational autoencoder. Prediction is performed to identify the class of the input image from 10 digit classes. Interpretation task involves attributing predictions made by this model to a subset of 28×28 (784) pixels. The attributions are computed on the joint network combining the encoder from the variational autoencoder and the digits feed-forward network. Additionally, we trained convolutional neural networks for handwritten digit recognition to demonstrate the usability of EIG on CNNs with and without an autoencoder. We trained two different CNNs for this task. First, we trained a standard CNN with convolutional, max-pooling layers followed by dense layers with ReLU activation function. Subsequently, we train a feed-forward neural network with convolutional variational autoencoder for dimensionality reduction. The input to the feed-forward neural network is the latent embedding from the convolutional variational autoencoder. Details about the architecture of the convolutional neural network can be found in Additional file 1: Table S7-S10.

### Evaluation of latent space stability

As is common in many genomic learning tasks, the splicing codes described above involve an embedding of the original features in a lower-dimensional latent space. This embedding leads to an extension of IG by using this latent space, but at the same time raises the question of whether the embedding itself is robust. Lack of robustness in embeddings can lead to undesirable scenarios where different network architecture choices lead to different interpretations. One way to ensure robustness of different embeddings is to ensure similar relative distances between different data points in different embeddings. Thus, we compute the Spearman rank correlation of the pairwise distances among training points in latent space between different autoencoders. We also evaluate correlations to the pairwise distances in the original feature space for comparison. Additional file 1: Fig. S3 shows that different autoencoder and variational autoencoder embeddings are stable (Spearman rank correlation between architectures ranges from 0.70 to 0.96).

### EIG: Enhanced Integrated Gradients

Integrated Gradients [8] is a feature attribution method for explaining predictions from a differentiable function *F* such as is obtained from deep learning models. The per-feature attributions for a prediction are defined relative to a reference point $\boldsymbol {x}^{\prime }\in \mathcal {X}$ and its prediction *F*(***x***^′^). For an observation $\boldsymbol {x}\in \mathcal {X}$, Integrated Gradients obtains an attribution vector ***attr***(***x***) by integrating the gradient of *F* with respect to the feature space along a path $\boldsymbol {\gamma }:[0,1]\rightarrow \mathcal {X}$ that starts at ***x***^′^ and ends at ***x***, i.e., ***γ***(0)=***x***^′^ and ***γ***(1)=***x***. If we write the attribution vector coordinate-wise as:

1 $$ \boldsymbol{\text{attr}}(\boldsymbol{x}) = [attr^{\boldsymbol{\gamma}}_{1}(x), attr^{\boldsymbol{\gamma}}_{2}(x), \ldots, attr^{\boldsymbol{\gamma}}_{p}(x)],  $$

then the attribution for the *j*th feature *x*_*j*_ is:

2 $$ attr^{\boldsymbol{\gamma}}_{j}(x) := \int_{\alpha=0}^{1}\frac{\partial F (\boldsymbol{\gamma}(\alpha))}{\partial \boldsymbol{\gamma}_{j}(\alpha)} \frac{\partial\boldsymbol{\gamma}_{j}(\alpha)}{\partial \alpha} d\alpha  $$

Sundararajan et al. [8] focuses on the special case where the path ***γ*** is chosen to take the straight-line path on $\mathbb {R}^{p}$ from ***x***^′^ to ***x***. Parameterized by *α*∈[0,1], the path is ***γ***(*α*)=***x***^′^+*α*(***x***−***x***^′^) so that the attribution for the *j*th feature is:

3 $$ attr_{j}(\boldsymbol{x}) := (\boldsymbol{x}_{j} - \boldsymbol{x}^{\prime}_{j}) \times \int_{\alpha=0}^{1} \frac{\partial F(\boldsymbol{x}^{\prime} + \alpha \times (\boldsymbol{x} - \boldsymbol{x}^{\prime}))}{\partial x_{j}}d\alpha  $$

To address the possibility of artifactual attributions by Integrated Gradients from linear paths crossing regions of $\mathbb {R}^{p}$ far from training points in feature space used to obtain *F*, we evaluate nonlinear paths designed to stay close to the data. We evaluate two approaches for generating such nonlinear paths: autoencoder networks on feature space and nearest neighbor graphs of training data. In the first approach, nonlinear paths are created by considering an autoencoder as the composition of an encoder $\mathcal {E}$ and decoder $\mathcal {D}$ to and from some latent space $\mathcal {L}$. We define $\boldsymbol {z}^{\prime } \equiv \mathcal {E}(\boldsymbol {x}^{\prime })$ and $\boldsymbol {z} \equiv \mathcal {E}(\boldsymbol {x})$. We take the linear path between ***z***^′^ and ***z*** and use $\mathcal {D}$ to map it back to a nonlinear path on $\mathcal {X}$. The final path accounts for the reconstruction error in mapping the endpoints back to $\mathcal {X}$ by connecting ***x***^′^ and ***x*** to the start and end of the decoded path (i.e., linearly interpolating between the endpoints and their auto-encoded counterparts).

In the second approach, nonlinear paths can be created in the original feature space $\mathcal {X}$ or the latent space $\mathcal {L}$. In $\mathcal {X}$, we construct the *k*-nearest neighbor graph on training data with respect to a distance metric on the $\mathcal {X}$, weighting edges by distances between points. The path between ***x***^′^ and ***x*** is created by adding them to the nearest neighbors graph and finding the shortest path between them using Dijkstra’s algorithm, interpolating linearly between neighbors. In $\mathcal {L}$, the procedure is similar except for two key differences. First, the distances are computed in $\mathcal {L}$. Second, the *k*-nearest neighbors path is computed between ***z***^′^ and ***z*** and the decoder $\mathcal {D}$ is used to map this path back to $\mathcal {X}$. 
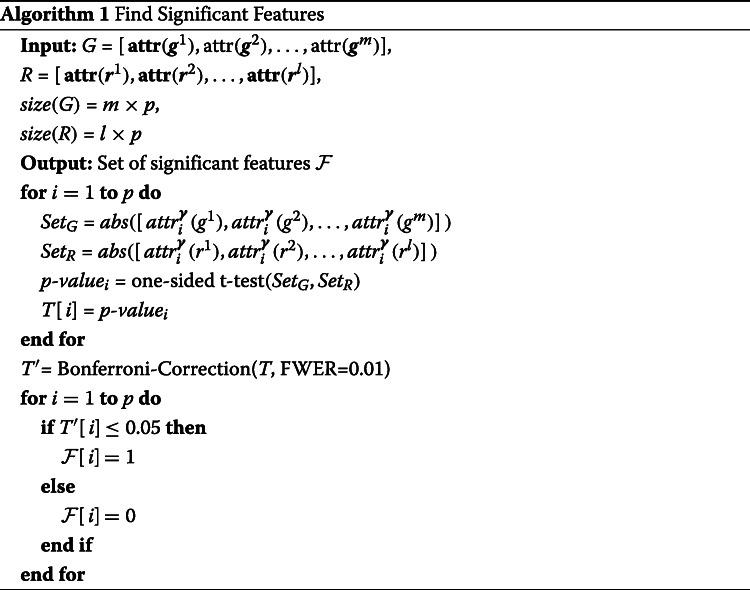


### Evaluation of IG-based methods for feature attribution

The original IG work by [8] only offered a method to compute the attribution per feature but did not offer a measure to identify significant features associated with a specific task. To address this need, we propose Algorithm 1. The input *G* contains attributions for all features/meta-features for a set of events of interest (e.g., differentially included exons in the brain), and *R* contains attributions for all features/meta-features for a random set of events. *Set*_*G*_ is a subset of *R* with absolute attribution for one feature/meta-feature for all examples from a class. *Set*_*R*_ is a subset of *G* with absolute attribution for one feature/meta-feature for a random subset of examples from all classes. For each feature/meta-feature, we perform a one-sided *t* test for the positive tail of the distribution on *Set*_*G*_ and *Set*_*R*_. The one-sided *t* test captures features where absolute attribution on *Set*_*G*_ is higher than *Set*_*R*_. To address the multiple-testing problem, we perform Bonferroni correction with family-wise error rate (FWER) of 0.01. A possible limitation of the above approach is that attribution may be dispersed between highly related features such as slight variations of the same splice factor binding motif. To address this issue in the context of splicing codes, we group highly correlated features into 781 meta-features, as done in previous works [18, 39]. To compute the attribution for a meta-feature, we sum the attributions of all its features. Then, we apply Algorithm 1 to find significant meta-features.

### Baselines

In the original IG work [8], an all-zero baseline is used to represent the absence of signal. This may be an acceptable choice for an object recognition task where it represents an all black image. However, in the genomics domain, an all-zero baseline may not be meaningful. As a first alternative, we propose a generic alternative baseline which we call encoded-zero. It requires an encoder/decoder to/from latent space such that we can use an all-zero point in the latent space and pass it to through the decoder to generate our baseline. The encoded-zero represents the mean of the data on which the autoencoder was trained. Interpretation with this baseline captures features that deviate from the mean and thus contribute to a sample’s prediction.

The encoded-zero baseline described above finds features that are important for a specific sample in comparison to the mean. However, in genomics, we are often interested in features that distinguish two classes of events. In order to distinguish the class of interest from a reference class, we propose four approaches to select one or more baseline points. *Random baseline*: in this approach, we randomly sample one or more points from the baseline class. This serves as the naive method to evaluate the effectiveness of the other methods of selecting baselines. *k-means baseline*: in this approach, we cluster the points of the baseline class to *k* different clusters and then use cluster centroids as baseline points. The number of clusters can be selected by cross-validation. For the splicing data, we found 3–5 clusters were sufficient (data not shown). This method gives baseline points that represent different subgroups that might be present in the baseline class. *Median baseline*: in this approach, we compute the euclidean distance of all the points of the baseline class from the median and select the points closest to the median. Points chosen using this method protect the later interpretation against outliers in the baseline class. *Close baseline*: in this approach, we compute the euclidean distance of all the points in the baseline class from all the points in the class of interest and pick points from the baseline class that are close to a sample from the class of interest as its baseline. These baseline points are close to the sample and may thus help capture a minimal set of distinguishing features between the baselines and the points of interest. When using this approach, we discard the closest point from the baseline class to avoid extreme outlier points.

### Defining the set of known biological features

As described in the main text, the set of splicing code features used in this work has been previously curated from the literature and is therefore highly enriched in informative splicing regulatory features in general, and for regulation of splicing in the muscle and brain in particular [18, 39]. To define a high-quality set of known biological meta-features from these for our specific task of interest, we therefore ran a hypergeometric test to compute *p* values for enrichment or depletion of a feature in the differentially included splicing events in the brain compared to a negative set of constitutive splicing events. To get a high-quality feature set, we used a highly conservative threshold of −*log*(*p*)>20 which resulted in several hundred features from those initially selected for the splicing code. Finally, we combined correlated features as meta-features and assigned the minimum *p* value assigned to a member of the meta-feature group as the *p* value for that meta-feature.

### Interpretation using Deep SHAP

Deep SHAP uses some points from a dataset as background. Given that we were comparing Deep SHAP with EIG using median-constitutive baseline, we used 1000 constitutive events as background. According to SHAP package that contains the Deep SHAP method, 1000 data points are sufficient for getting very good estimate of the expected values for the background [20]. The set of interest was similar to EIG, differentially included cassette AS events in mouse hippocampus in comparison to five other tissues.

### Analysis of liver alternative events

To focus on high confidence, liver alternative cassette exons, we focused our analysis on 243 cassette exon events that were deferentially included when comparing the liver with two or more of the other five tissues. These exons include the set of exons with increased inclusion in the liver or increased exclusion in the liver versus at least two other tissues. The alternative in other tissue set was defined as the 382 cassette exons that were deferentially included in two or more pairwise comparisons among the five non-liver tissues that also did not change in any liver comparisons. The non-changing in liver set was defined as the 8426 events that were not changing in any of the comparisons of the liver with the other five tissues.

### Analysis of A1CF RNA-seq

We processed RNA-seq data from wild-type and *A1cf* knockout mouse livers [32] using MAJIQ [17] to quantify splicing and identify significant changes in splicing between these groups (|*ΔΨ*|≥10*%*). We matched these significant changing junctions to the cassette exons defined above and defined 171 A1CF-regulated exons to be all cassettes with both an inclusive and exclusive junction with |*ΔΨ*|≥10*%*. This set of regulated exons included A1CF enhanced exons (i.e., decreased inclusion upon *A1cf* knockout compared to wild-type) and A1CF repressed exons (i.e., increased inclusion upon *A1cf* knockout). A1CF non-regulated exons were defined as the 2748 events with all junctions in the cassette exon or the broader local splicing variation (LSV) event as defined by MAJIQ [17] changing with |*ΔΨ*|<5*%*.

### Motif analysis around cassette exons

We extracted sequences for cassette exons and the splice site proximal intronic regions upstream and downstream of the intron. For each subset of cassette exon events, we calculated the frequency of A1CF motif occurrence at each position (defined to be AAU, UAA, or AUU [29, 34]). For plotting, the frequencies were smoothed using a running mean of 20 nucleotides. To find regions of significant difference between sets of cassette exons, we searched for the motif over a sliding window of 20 nucleotides and performed Fisher’s exact test comparing the number of events containing a motif in the regulated (e.g., deferentially included in the liver) compared to non-regulated (e.g., non-changing in the liver) exon sets at each window. We reported positions with a two-tailed *p* value <0.05 and displayed a − log10 transformation of the *p* values on the plots.

### A1CF PAR-CLIP analysis

We processed PAR-CLIP data from the mouse liver from [32] using the CLIP Tool Kit (CTK) [35] using the recommended protocol for PAR-CLIP data. Briefly, reads were assessed for quality, adapters were trimmed, and exact PCR duplicates were collapsed. Trimmed and collapsed reads were aligned using BWA [40] with the options -t 4 -n 0.06 -q 20. After another round of PCR duplicate collapsing, CTK was used to identify unique tags containing the T-to-C transitions. Data from the duplicate experiments were merged, and we retained the transition sites identified by two or more unique tags for downstream analysis. We searched for the presence of such tags around the various exon sets defined above either within the alternative exon, within 300 nucleotides upstream of the alternative exon, within 300 nucleotides downstream of the alternative exon, or within any of the three aforementioned regions. Significance between regulated and non-regulated exon sets for A1CF binding was assessed using a two-tailed Fisher’s exact test.

### Path interpolation implementation

Paths are approximated discretely by a sequence of points on the path. Integration is performed numerically using the trapezoidal rule as implemented in NumPy. Approximation error of integration is estimated in two ways: (1) by comparing the sum of attributions to the difference between the predictions at the endpoints (as suggested by [8]) and (2) by halving the step size for numerical integration to evaluate relative agreement between more refined approximations. Estimated integration error is used to determine the number of points necessary in the discrete paths (see Additional file 1: Fig. S4).

## Supplementary information

**Additional file 1** Supplementary information, supplementary tables S1–10, and supplementary figures S1–12.

**Additional file 2** Review history.

## Data Availability

Mouse tissue RNA-seq data (MGP) used for this analysis is available in ArrayExpress under accession E-MTAB-599 [41]. RNA-seq data from the livers of wild-type and *A1cf* knockout mice and the A1CF PAR-CLIP data are available in NCBI Sequence Read Archive (SRA) under accessions PRJNA530736 and PRJNA531626, respectively [42, 43]. MNIST digit data is available at [10]. Source code for all analyses, results, and figures has been deposited to Zenodo [44], under a BSD 3-clause license. The EIG library and resources can be viewed and downloaded at: https://bitbucket.org/biociphers/eig/.
