# Supplementary material for: Enhanced Integrated Gradients: improving interpretability of deep learning models using splicing codes as a case study
Source: Genome Biol. 2020 Jun 19;21:149. doi: 10.1186/s13059-020-02055-7 (PMC7305616; doi:10.1186/s13059-020-02055-7)
Supplement: Supplementary file 1 — Additional file 1 Supplementary information, supplementary tables S1–10, and supplementary figures S1–12. [file 13059_2020_2055_MOESM1_ESM.pdf]

SUPPLEMENTARY INFORMATION FOR ENHANCED INTEGRATED GRADIENTS: IMPROVING INTERPRETABILITY OF DEEP LEARNING MODELS USING SPLICING CODES AS A CASE STUDY

Anupama Jha[1], Joseph K. Aicher[2*], Matthew R. Gazzara[2*], Deependra Singh[1] & Yoseph Barash[1,2]
[1]Department of Computer and Information Science, School of Engineering
[2]Department of Genetics, Perelman School of Medicine
University of Pennsylvania, Philadelphia, PA 19104, USA
[*]Equal contribution
Correspondence to: `yosephb@upenn.edu`

S1 PLOTTING DETAILS FOR REAL PATHS ON SPLICING DATA (FIG 1A, BOTTOM PANEL)

The plot shows PC1 (horizontal axis) versus PC2 (vertical axis) of original feature space ($1,357$ features) trained on splicing data. The scattered gray points are subset of input data in the PC space. Black, red, blue paths show linear, latent-linear, and neighbors paths between the same source and destination points (matching conceptual/illustrative figure 1a, top panel). Source point is picked randomly and the destination point is picked to maximize distance from source point in PC space (10 components). Neighbors path is approximation, i.e., computing neighbors distances on first 10 principal components due to computational overhead. Random seeds were manually selected to highlight differences between linear and latent linear paths.

S2 PATH INTERPOLATION

The number of points used to interpolate paths between a sample and a baseline was selected by estimating the relative numerical integration error for paths between randomly sampled pairs of points. **Relative error** was estimated in two ways: (1) **sum error**, and (2) **median reinterpolation error**.

Sum error is described in (1) and uses the property:

$$\begin{aligned}\sum \text{attributions}(f, \gamma[a \to b]) &= \sum \int_{\gamma[a\to b]} \nabla f \, \mathrm{d}\gamma \\ &= \int_{\gamma[a\to b]} \sum \nabla f \, \mathrm{d}\gamma \\ &= \int_{\gamma[a\to b]} \nabla f \cdot \mathrm{d}\gamma \\ &= f(b) - f(a)\end{aligned}$$

We define the sum error as the relative difference between the left-hand-side and the right-hand-side of the equation, using the numerically calculated attributions for the left hand side. Specifically:

$$\text{sum error (relative)} = \frac{|\text{RHS} - \text{LHS (numerical)}|}{\varepsilon + |\text{RHS}|}.$$

Median reinterpolation error is obtained by comparing attribution estimates from a path with the given number of points to a refined path with additional points interpolating between the original points. For a given feature, we say that g^* is the true attribution which is being estimated numerically

by $g(n)$ for a path with n points. Then, reinterpolating with r points, we estimate the relative error for the feature attribution as:

$$2 \times \frac{|g(n) - g(rn)|}{\varepsilon + \max(|g(n)|, |g(rn)|)}.$$

This gives a relative error estimate per feature for a path between points. The median reinterpolation error is the median of these estimates across features.

We calculated these estimates for 200 pairs of randomly selected points in our dataset using linear paths, comparing 50, 100, 250, 500, and 1000 points in the path. We found that 51% of the replicates have zero relative error regardless of estimation method or number of points, and 2% of the replicates have outlying sum errors greater than 10^5 due to a negligible difference between predictions for the source and destination points. Plots of relative error (sum error and median interpolation error) are found in Supplementary Figure S4. We decided to use 250 points for our subsequent experiments as it had an acceptable relative error.

REFERENCES

[1] Sundararajan, M., Taly, A. & Yan, Q. Axiomatic attribution for deep networks. *arXiv:1703.01365 [cs]* (2017). URL `http://arxiv.org/abs/1703.01365`. `1703.01365`.

[2] Ray, D. *et al.* A compendium of rna-binding motifs for decoding gene regulation. *Nature* **499**, 172 (2013).

[3] Dominguez, D. *et al.* Sequence, structure, and context preferences of human rna binding proteins. *Molecular cell* **70**, 854–867 (2018).

Table S1: **Splicing Code Autoencoder(AE) and Variational Autoencoder(VAE) Architecture**

Name	Hidden units and layers	Activation Function
AE200	1357, 850, 500, 200, 500, 850, 1357	tanh
AE400	1357, 850, 500, 400, 500, 850, 1357	tanh
AE500	1357, 850, 500, 850, 1357	tanh
AE600	1357, 850, 600, 850, 1357	tanh
VAE100	1357, 850, 500, 100, 500, 850, 1357	tanh
VAE200	1357, 850, 500, 200, 500, 850, 1357	tanh
VAE500	1357, 850, 500, 500, 500, 850, 1357	tanh

Table S2: **Splicing Code Feed Forward Network Architecture**

Name	Hidden units and layers	Activation Function
AE500-encoded Network	500, 500, 200, 50, 6	ReLU

Table S3: **Splicing Code Combined Encoder Feed Forward Network Architecture**

Name	Hidden units and layers	Activation Function
Combined AE500-encoded Network	1357, 850, 500, 500, 200, 50, 6	ReLU

Table S4: **MNIST Digit Variational Autoencoder(VAE) Architecture**

Name	Hidden units and layers	Activation Function
VAE50	784, 500, 500, 50, 500, 500, 784	ELU, tanh

Table S5: **MNIST Digit Feed Forward Network Architecture**

Name	Hidden units and layers	Activation Function
VAE50-encoded Network	50, 400, 200, 50, 10	ReLU

Table S6: **MNIST Digit Combined Encoder Feed Forward Network Architecture**

Name	Hidden units and layers	Activation Function
Combined VAE50-encoded Network	784, 500, 500, 50, 400, 200, 50, 10	ELU, tanh, ReLU

Table S7: **MNIST Digit Convolutional Neural Network Architecture**

Layer Type	Dimensions	Activation Function
Conv2D	filters: 32, filter size: (3, 3)	ReLU
Conv2D	filters: 32, filter size: (3, 3)	ReLU
MaxPool2D	Pool size: (2, 2)	None
Dropout	Rate: 0.2	None
Conv2D	filters: 32, filter size: (3, 3)	ReLU
Conv2D	filters: 32, filter size: (3, 3)	ReLU
MaxPool2D	Pool size: (2, 2)	None
Dropout	Rate: 0.25	None
Conv2D	filters: 128, filter size: (3, 3)	ReLU
Dropout	Rate: 0.25	None
Flatten	None	None
Dense	hidden units: 128	ReLU
Batch Normalization	None	None
Dropout	Rate: 0.25	None
Dense	Number of Labels: 10	Softmax

Table S8: **MNIST Digit Convolutional Variational Autoencoder Architecture**

Layer Type	Dimensions	Activation Function
Conv2D	filters: 32, filter size: (3, 3), strides: (2, 2)	ReLU
Conv2D	filters: 64, filter size: (3, 3), strides: (2, 2)	ReLU
Flatten	None	None
Dense (latent mu)	hidden units: 50	None
Dense (latent sigma)	hidden units: 50	None
Dense	hidden units: 1568	ReLU
Conv2D-transpose	filters: 64, filter size: (3, 3), strides: (2, 2)	ReLU
Conv2D-transpose	filters: 32, filter size: (3, 3), strides: (2, 2)	ReLU
Conv2D-transpose	filters: 1, filter size: (3, 3), strides: (1, 1)	ReLU

Table S9: **MNIST Digit Feed-Forward Architecture**

Layer Type	Dimensions	Activation Function
Dense	hidden units: 400	ReLU
Dropout	Rate: 0.25	None
Dense	hidden units: 200	ReLU
Dropout	Rate: 0.25	None
Dense	hidden units: 50	ReLU
Dropout	Rate: 0.25	None
Dense	Number 0f Labels: 10	Softmax

Table S10: **MNIST Digit Convolutional Feed-Forward Architecture**

Layer Type	Dimensions	Activation Function
Conv2D	32 filters, filter size: (3, 3), strides: (2, 2)	ReLU
Conv2D	64 filters, filter size: (3, 3), strides: (2, 2)	ReLU
Flatten	None	None
Dense (latent mu)	50	None
Dense	400	ReLU
Dropout	Rate=0.25	None
Dense	200	ReLU
Dropout	Rate=0.25	None
Dense	50	ReLU
Dropout	Rate=0.25	None
Dense	Number 0f Labels = 10	Softmax

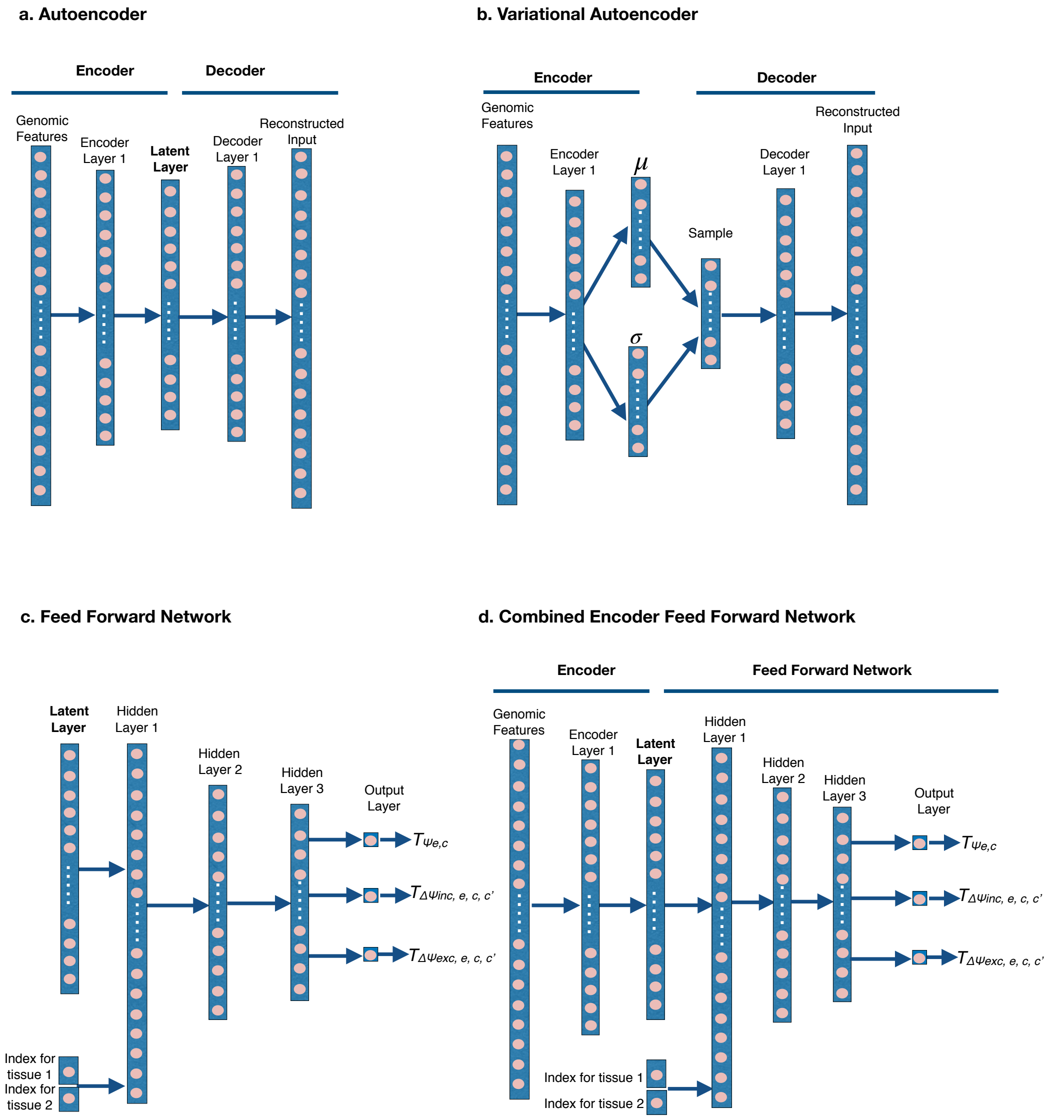

Fig S1: **Splicing code architecture.** **a.** Illustration of the autoencoder architecture for the splicing code. **b.** Illustration of the variational autoencoder architecture for the splicing code. The latent layer learns mean μ and standard deviation σ of the Gaussian distribution from which samples are drawn for the decoder. **c.** Illustration of the feed forward network for the splicing code. The latent layer from **a** is input to the model. The output of the model contains three targets: $\mathrm{T}_{\Psi_{e,c}}$ is the expected PSI value of the event e in condition c, $\mathrm{T}_{\Delta\Psi_{inc,e,c,c'}}$ captures the dPSI for event e if it has increased inclusion between condition c and c' and $\mathrm{T}_{\Delta\Psi_{exc,e,c,c'}}$ captures the dPSI for event e if it has increased exclusion between condition c and c'. **d.** Combined network from the encoder of the autoencoder in **a** and the feed forward network from **c**.

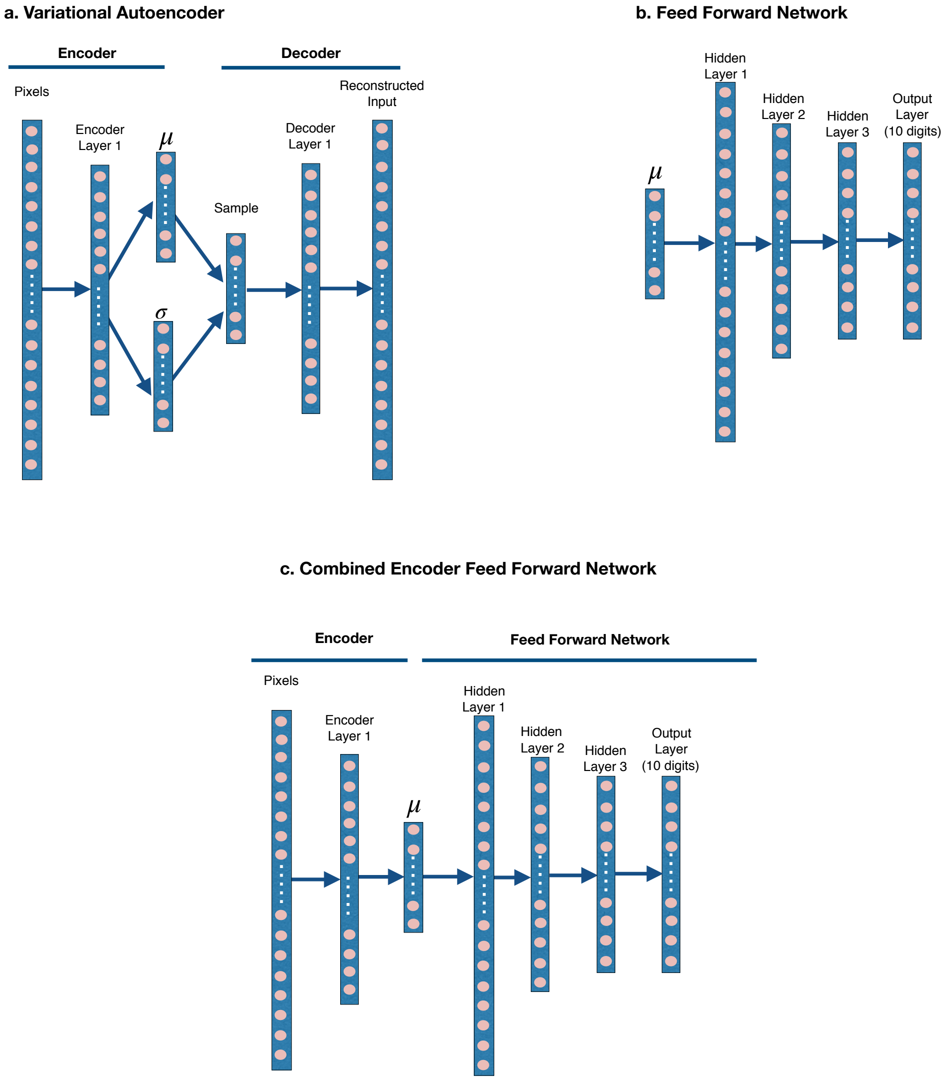

Fig S2: **MNIST handwritten digit architecture. a.** Illustration of the variational autoencoder architecture for the digits model. The latent layer learns mean μ and standard deviation σ of the Gaussian distribution from which samples are drawn for the decoder. **b.** Illustration of the feed forward network for MNIST handwritten digit task. The mean layer μ from **a** is input to the model. The output is a softmax over 10 digits. **c.** Combined network from the encoder of the variational autoencoder in **a** and the feed forward network from **b**.

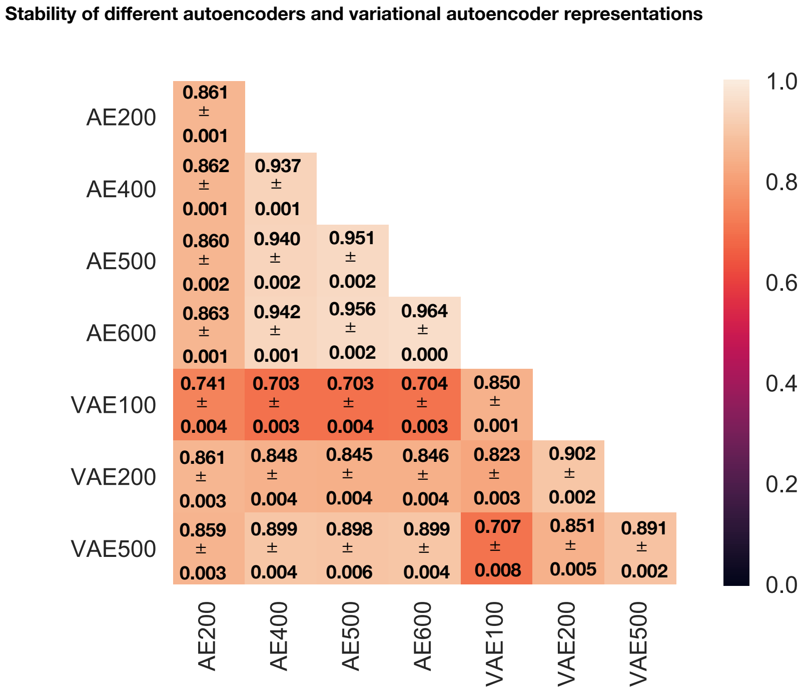

Fig S3: **Stability analysis of autoencoders and variational autoencoders for the splicing code.** Spearman rank correlation of the pairwise distances among training points in latent space between different autoencoders and variational autoencoders. The distances are calculated for three randomly selected subset of training points to calculate the standard error. Supplementary Table S1-S6 describe the architecture of these networks.

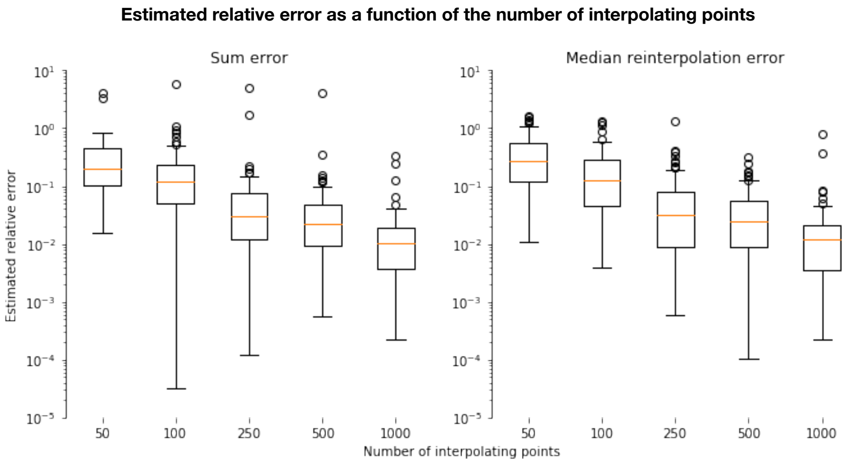

Fig S4: **Estimated relative error as a function of the number of interpolating points**. The plots show relative error, as estimated by **(left)** sum error and **(right)** median reinterpolation error, versus an increasing number of points used to create a path between points. The relative error distributions are estimated over 200 pairs of randomly sampled points in the data, interpolated by each of the described number of points. 51% of the replicates have zero relative error regardless of estimation method or number of points and are excluded from the plot. 2% of the replicates have outlying sum errors greater than 10^5 due to a negligible difference between predictions for the source and destination points and are excluded from the plot.

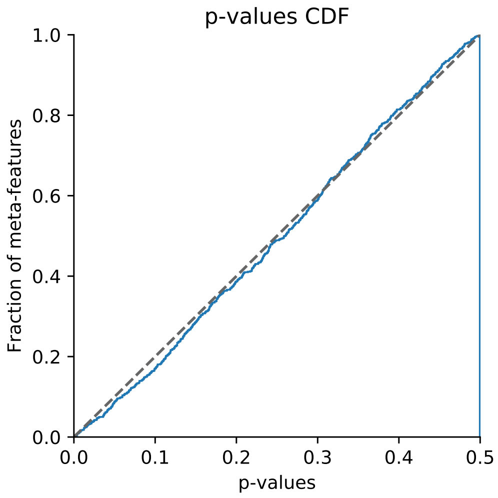

Fig S5: **Calibration of *p*-value distribution**. Cumulative distribution of p-values for all meta-features using two random group of splicing events (one-sided t-test; $n = 781$ pairwise comparisons). The distribution of p-values is close to random (diagonal), indicating that empirical distribution of p-values is well calibrated.

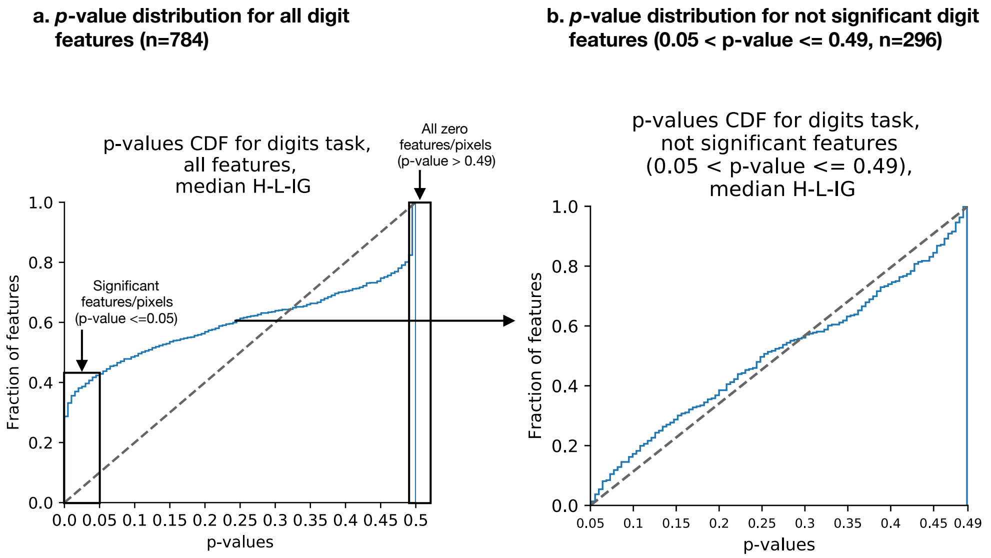

Fig S6: ***p*-value distributions for digit features with median H-L-IG**. Cumulative distribution of p-values for: **a.** all pixel features, **b.** After removing significant pixel features and "dead" pixels that are always black background ($0.05 < p$-values ≤ 0.49) using samples from class of interest (samples: 5, baseline: 3) and a random background group (samples: $1, 5, 7$, baseline: 3). One-sided t-test; $n = 784$ pairwise comparisons.

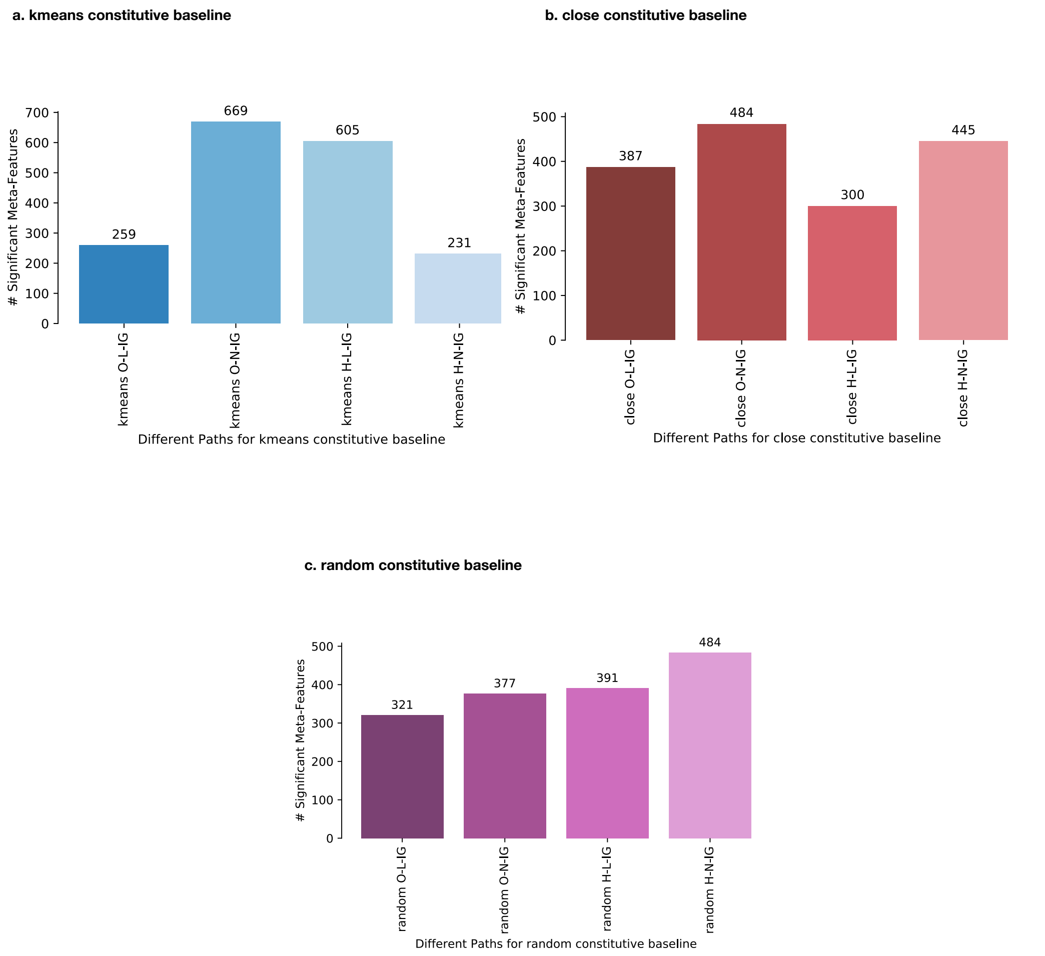

Fig S7: **The effect of different paths and baselines on the number of significant meta-features identified. a.** Number of significant meta-features identified by different paths with three kmeans-constitutive baseline points. **b.** Number of significant meta-features identified by different paths with three closest-constitutive baseline points. **c.** Number of significant meta-features identified by different paths with three random-constitutive baseline points.

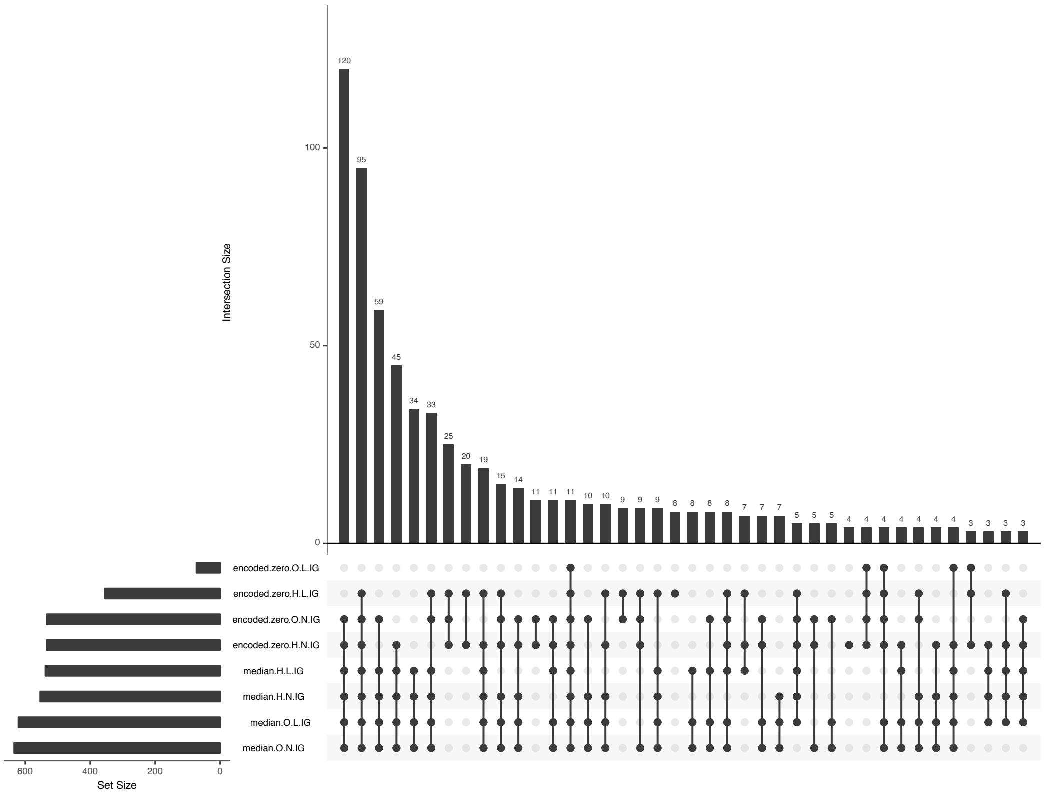

Fig S8: **Overlap of significant meta-features found by encoded-zero and median baselines.** The plot shows various intersections of meta-features identified as significant using O-L-IG, O-N-IG, H-L-IG and H-N-IG paths for encoded-zero and median-constitutive baselines.

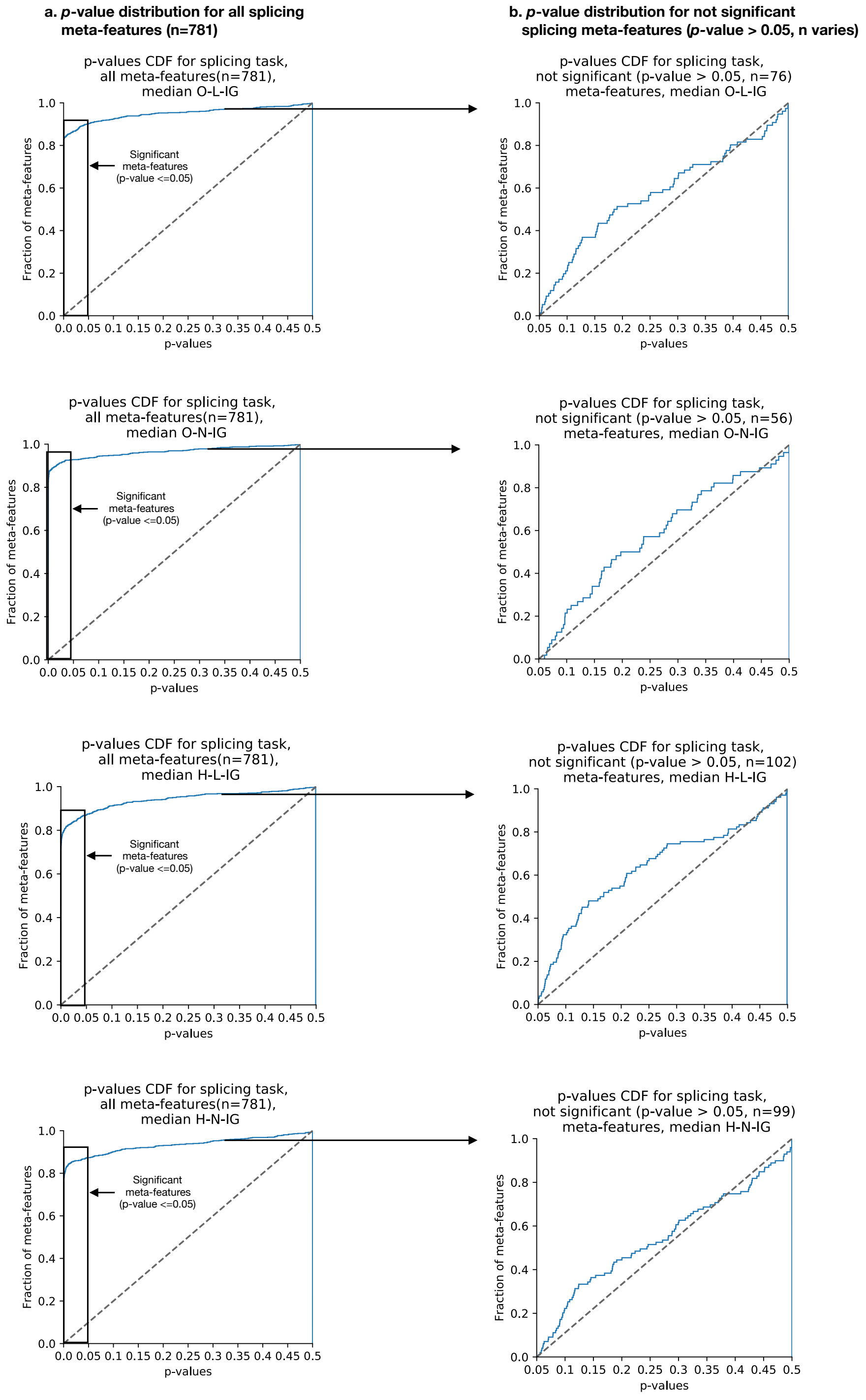

Fig S9: ***p*-value distributions for splicing meta-features with median baseline**. Cumulative distribution of *p*-values for all meta-features using differentially included splicing events as one class and random group of splicing events as the other. **a.** all meta-features and **b.** not significant meta-features (*p*-value > 0.05). One-sided t-test; $n = 781$ pairwise comparisons. Unlike in the digit prediction task, we observe here somewhat of an inflation of *p*-values compared to random even after the removal of the informative feature set. This is likely the result of the original definition of the feature set, curated from the literature and carrying relevant information for splicing regulation. For example, tri-nucleotide frequencies can still capture partial binding motifs removed in the informative feature set.

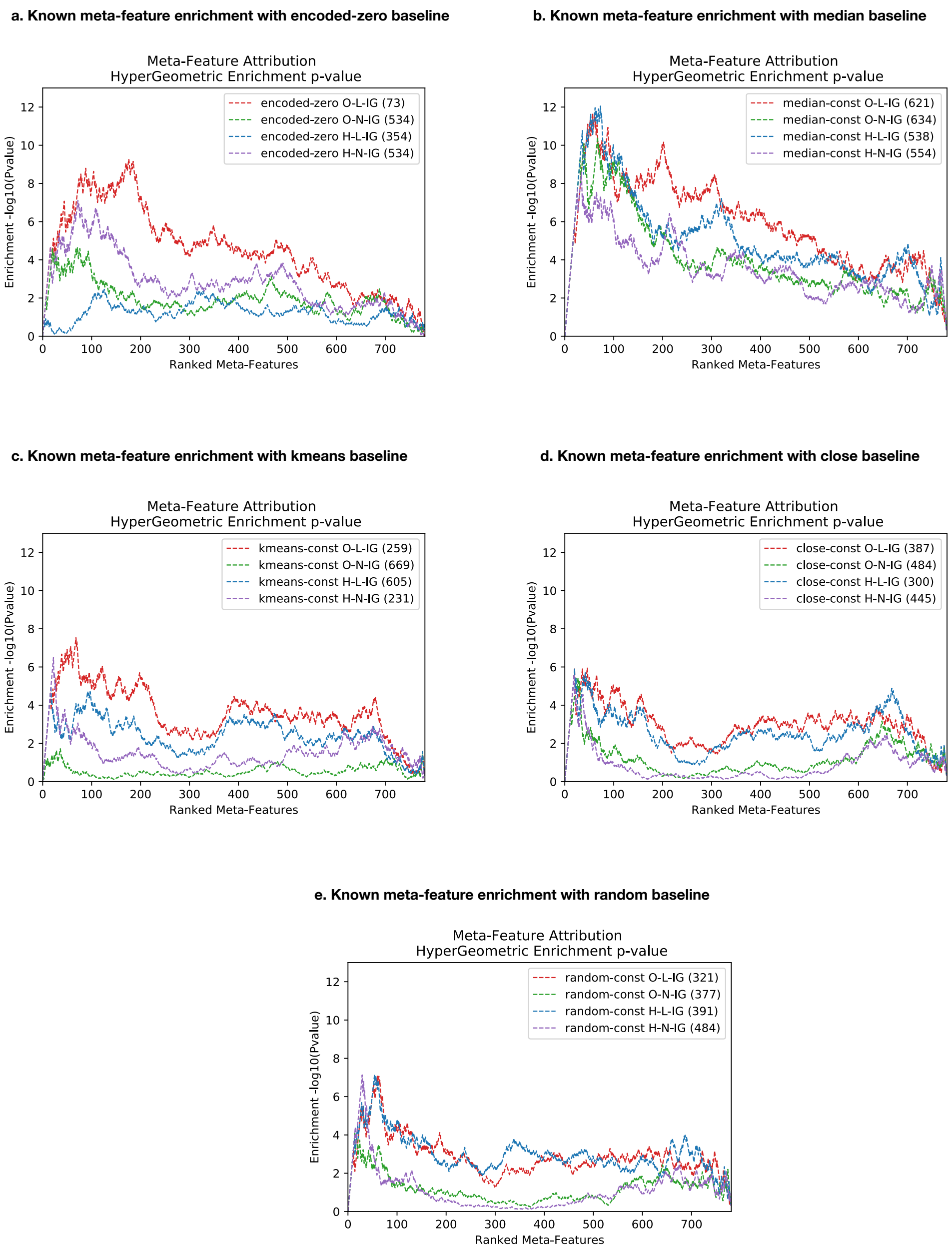

Fig S10: **Enrichment of known biological features with EIG baselines and paths**. Enrichment of known brain regulatory features in significant features identified by EIG paths with **a.** encoded-zero baseline, **b.** median baseline, **c.** kmeans baseline, **d.** close baseline, and **e.** random baseline.

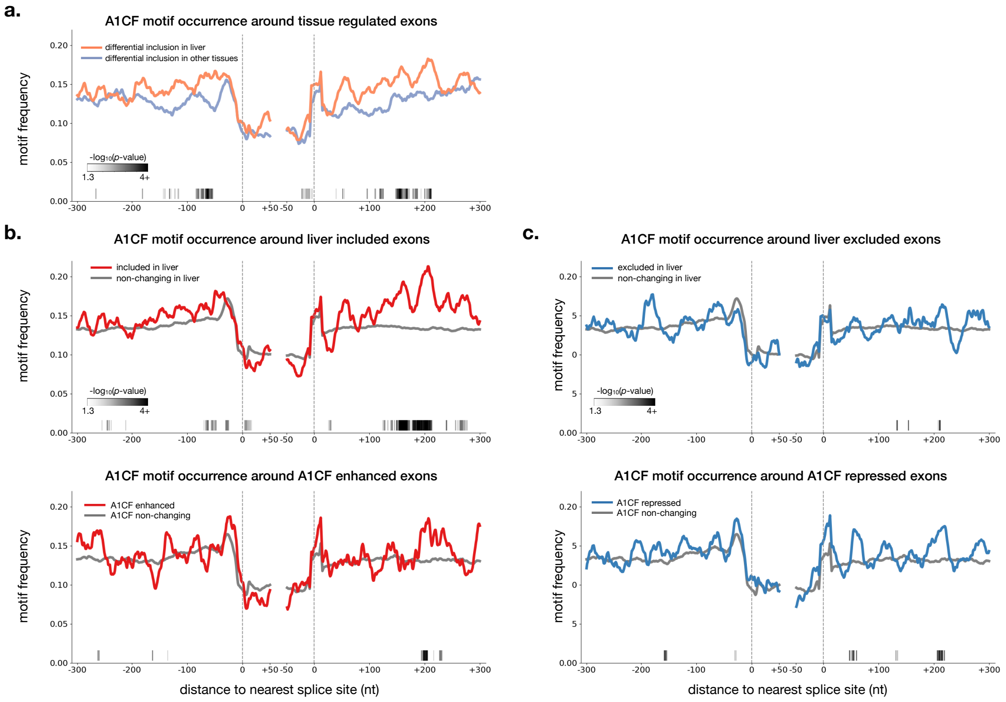

Fig S11: **A1CF motif analysis around regulated exon sets. a.-c.**, Motif maps showing the frequency of 3-mers known to bind A1CF (AAU, UAA, or AUU (2), (3)) around the 3' and 5' splice sites of cassette exon sets indicated in the legends. Frequencies were smoothed using a running mean of 20 nucleotides (nts). Grey scale boxes indicate significant differences in motif occurrence ($-\log_{10}(p)$) between the regulated versus non-regulated exon sets (Fishers exact test assessed at sliding windows of 20 nts).

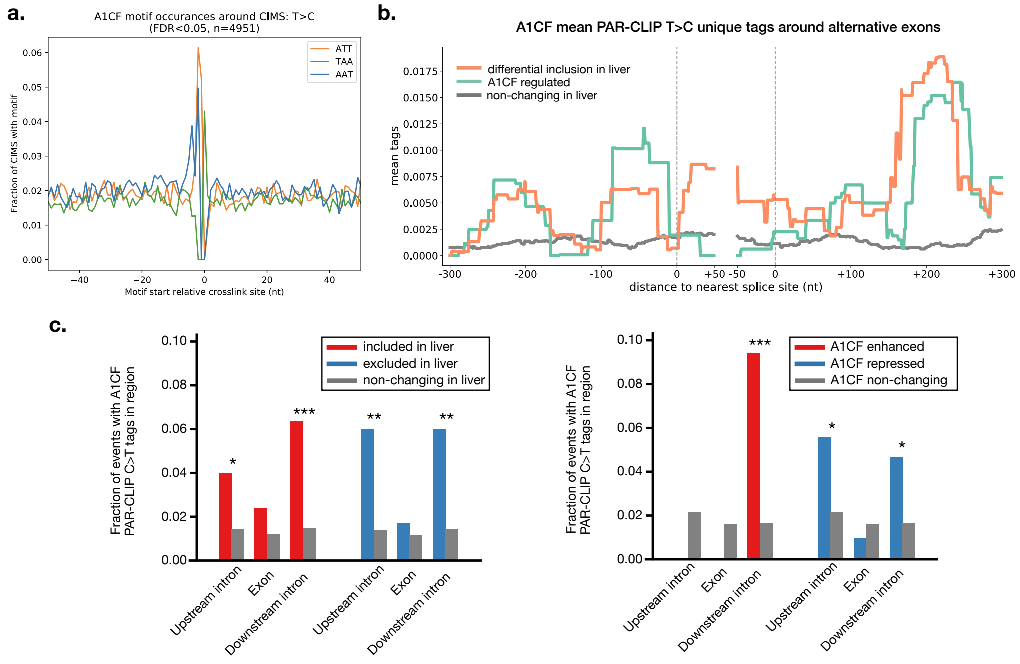

Fig S12: **A1CF PAR-CLIP analysis**. **a.** Frequency of motif start positions of the 3-mers known to bind A1CF (AAU, UAA, or AUU (2), (3)) relative to the T-to-C transition site in the PAR-CLIP tags. **b.** RNA map showing the mean number of unique T-to-C transition PAR-CLIP tags at positions around the 3' and 5' splice sites of cassette exon sets indicated in the legends. Data was smoothed using a running mean of 75 nucleotides. **c.** Barchart displaying fraction of alternative exon events in the indicated sets which contained T-to-C transition PAR-CLIP tags in the indicated region around the alternative exon. These regions are: within 300 nt upstream of the alternative exon 3'SS (Upstream intron); within the alternative exon (Exon); or within 300 nt downstream of the alternative exon 5'ss (Downstream intron). Asterisks indicate significant differences between the regulated (red or blue) versus non-regulated (gray) exons sets (Fisher's Exact two-tailed $p < 0.05$ (*), $p < 0.01$ (**), or $p < 0.001$ (***))
