# Supplementary material for: Enhanced Integrated Gradients: improving interpretability of deep learning models using splicing codes as a case study
Source: Genome Biol. 2020 Jun 19;21:149. doi: 10.1186/s13059-020-02055-7 (PMC7305616; doi:10.1186/s13059-020-02055-7)
Supplement: Supplementary file 2 — Additional file 2 Review history. [file 13059_2020_2055_MOESM2_ESM.docx]

**Response to reviewers**

**1st round**

**Reviewer 1**

The utility of deep learning for genomic data hinges on the ability to interpret the resulting models. The authors propose several improvements over the integrated gradients method that also makes it more applicable for genomic data. The method is tested on two tasks - image classification and splicing code analysis. Follow up analysis demonstrates the ability of the method to shed light on splicing regulation. The method is well thought out and potentially very useful. As currently presented however, its usefulness may be limited as described below.

Comments

1. IG is a commonly used method for feature attribution, i.e. determining the relevant features for the classification of a given sample. The authors' latent integrated gradients (LIG) addresses several issues in IG that are reported to improve its performance, and make it applicable for genomic data.

2. The primary limitation of IG cited by the authors is that it only performs feature attribution with respect to a particular sample. I don't see that as a limitation - it does what it was designed to do, and feature attribution is often what's needed for a biologist to determine the biological signal that is relevant to the classification of a given sample. Their extension of IG make it a feature scoring method, but I don't see that as the primary contribution.

3. The authors test the method on deep learning tasks that address data in a fixed-dimensional feature space. However, perhaps the most common use of deep learning, and where its primary contribution lies, is in the context of convolutional or recurrent networks. The original IG method is applicable in this context, and has been tested for a variety of architectures, but the authors have not attempted to show the usability of their method for more interesting architectures. It's not clear that the method is applicable in such cases because of the reliance on a latent space in which to perform the integration (how about using the activations of fully connected layers after convolution has been applied?). If that is indeed the case, it should be pointed out in the manuscript, as it would severely limit the usability of the method. The authors have studied splicing using CNNs, and it would be interesting to see what they can find using LIG for that data, if it can be used. At the very least, some of the improved baselines would likely be useful.

4. It's not clear that an autoencoder is required to apply the method - one can view the hidden neurons of a network trained using supervised learning as an embedding instead of having to perform an additional step of training an autoencoder. Please comment on that.

5. The authors use the number of significant features that a method discovers as indication of its quality. However, in the context of feature selection, the goal is typically to find a small set of features that provide accurate performance. Admittedly, the finding that their LIG method finds significant features where the original IG method does not, is an indication of better performance, it's difficult to make a case that finding say 500 is better than 200, and the authors admit that.

6. Summing up the contributions of features that are part of a meta-feature is not a good idea in my opinion - it provides an inordinately large weight for such features, and make it such that they are more likely to be selected compared to others. Taking the maximum of a cluster of features would be more appropriate.

Minor comments

1. In some areas the Results section is not clear - mentioning methods or terms that have not been discussed (downside of a Results-first paper organization). For example, "our autoencoder" is mentioned before the reader has any notion how those were used. Similarly, at the paragraph at the top of page 3, second column, more background is required to understand the discussion.

2. It was not clear if feature selection was performed on the training set only. Please clarify that.

3. The nearest neighbor path method doesn't seem to perform as well and is more computationally expensive. Consider removing it to improve the readability of the manuscript (could be mentioned that such a method was considered, but did not perform at the same level as the other proposed variations). The results section presents many variants that make it challenging to keep track of all of them.

4. Set_R and Set_G are not defined in the text and are not really needed outside of Algorithm 1. Algorithm 1 itself needs to be more clear (e.g. what are G and R).

5. k-means and median baseline - in which space are these computed?

6. You mention that you analyzed "a significant meta-feature identified through this procedure" - exactly which procedure are you referring to? LIG has several flavors. Were all of them able to detect it? What made you choose that particular meta-feature?

Grammar/notation/references

- Ref 9 was published in ICML.

- Check capitalization of references (e.g. ref 15)

- "First the distances are computed on the L" --> are computed in L or are computed in the latent space.

- linear paths in original and latent space --> the original and latent spaces

**Reviewer 2**

The manuscript by Jha et al. describes strategies to evaluate feature importance for deep learning models using integrated gradients (IG). This work addressed several limitations of the original IG method: 1) selection of reference, as a zero is frequently not meaningful for genomic data; 2) the path of integration from reference to the sample; 3) a significance test to evaluate the importance of features related to the classification task instead of a specific sample. These improvements are very useful for effective interpretation of deep learning models in many potential applications. The authors demonstrated effectiveness of the proposed improvements in digit application and tissue-specific alternative splicing prediction. Interestingly, the authors found A1CF as a regulator of liver specific splicing. Overall, this manuscript provides very useful insights into an important topic. However, the manuscript can be improved in many ways for rigor and clarity.

1. The authors uses splicing code as a major application, and mentioned several RBPs such as Rbfox and Nova. It will be helpful to provide some introduction of these RBPs and other features used for prediction.

2. P3, The authors clustered original features into meta-features. How many meta-features after grouping correlated features?

3. P3, in the section, "IG with nonlinear paths identify significant features missed using linear paths or simple gradients". The authors should explicitly state that the results are from zero baseline in the original feature space.

4. Baseline group and group of interest should be defined more clearly.

5. P4: calibration of p-values using random group of events. It will also be helpful to plot the distribution of p-values in the real dataset.

6. P4, enrichment of known biological features - details are missing completely. Which features are "known biological features"?

7. P4, "Identified significant features improve splicing prediction" - not clear what does that mean from the comparison of 70 meta features vs 142 original features. It will be more informative by comparison of significant features vs. a set of randomly selected features or a set of features selected by a baseline method of equal size.

8. Fig. 3a, poor visibility of the images with background.

9. Fig. 4 cd, the authors interpreted some of the peaks as biological important. Overall, the RNA map in panel c is quite noisy, and it is unclear peaks are real or due to random fluctuations. It will be helpful to distinguish liver-specific exons by liver-specific inclusion/skipping, and similarly A1CF dependent exons by activation/repression, and then analyze motif enrichment in exon, upstream/downstream introns separately.

10. Fig. S1: label. It is weird to have "genomic features" and "tissue" for a digit classification task. The labels should be corrected.

11. Fig. S2: 2b, encoder layer 1 -> decode layer 1

**Response to reviewers**

We first want to thank the reviewers for their thoughtful and constructive feedback. We believe we have addressed all of the comments/suggestions made, and thanks to this constructive feedback the manuscript has been significantly improved. We hope you find the revised work suitable for publication. We address the questions and concerns raised by the reviewers point by point below. We also include an annotated PDF where the majors areas of changes are marked and comments were added to refer each change to the various reviewers’ comments.

Reviewer #1: (Remarks to the authors)

The utility of deep learning for genomic data hinges on the ability to interpret the resulting models. The authors propose several improvements over the integrated gradients method that also makes it more applicable for genomic data. The method is tested on two tasks - image classification and splicing code analysis. Follow up analysis demonstrates the ability of the method to shed light on splicing regulation. The method is well thought out and potentially very useful. As currently presented however, its usefulness may be limited as described below.

Comments

1. IG is a commonly used method for feature attribution, i.e. determining the relevant features for the classification of a given sample. The authors' latent integrated gradients (LIG) addresses several issues in IG that are reported to improve its performance, and make it applicable for genomic data.

2. The primary limitation of IG cited by the authors is that it only performs feature attribution with respect to a particular sample. I don't see that as a limitation - it does what it was designed to do, and feature attribution is often what's needed for a biologist to determine the biological signal that is relevant to the classification of a given sample. Their extension of IG make it a feature scoring method, but I don't see that as the primary contribution.

We very much agree that IG can be used to find relevant biological signals/features with respect to a sample and if one is interested in a particular sample, that might be sufficient. What we point out is a lack of framework when it comes to identifying features significant for a class of samples. In its present form, one needs to do post hoc processing with the attributes generated with IG in order to conclude anything about biological signals relevant to a class of sample (e.g. ranking by average attributes across samples). We propose the significance framework as an alternative to this post hoc processing if one needs to answer questions about class-wide biological signals/features.

3. The authors test the method on deep learning tasks that address data in a fixed-dimensional feature space. However, perhaps the most common use of deep learning, and where its primary contribution lies, is in the context of convolutional or recurrent networks. The original IG method is applicable in this context, and has been tested for a variety of architectures, but the authors have not attempted to show the usability of their method for more interesting architectures. It's not clear that the method is applicable in such cases because of the reliance on a latent space in which to perform the integration (how about using the activations of fully connected layers after convolution has been applied?). If that is indeed the case, it should be pointed out in the manuscript, as it would severely limit the usability of the method. The authors have studied splicing using CNNs, and it would be interesting to see what they can find using LIG for that data, if it can be used. At the very least, some of the improved baselines would likely be useful.

We thank the reviewer for this extremely relevant feedback. We have taken several steps to address the reviewer concerns that are listed below.

- First, to better frame both the method and the contributions of this work we renamed the method from LIG (latent integrated gradients) to EIG (enhanced integrated gradients). We think the term LIG was not appropriately conveying the improvements added to the original IG method and led to some of the (rightful!) confusions expressed by the reviewer. Specifically, EIG is not just IG in latent spaces but rather three main improvements to the IG method: improved baselines, linear and non-linear path definitions both in original and latent spaces and the feature significance framework and EIG conveys this better.

-To show that EIG works on other network architectures we trained a CNN on the task of handwritten digit recognition and show it performs well (Figure 2b). While we did not try to add an RNN (we think it does not make much sense on this task) we added a short discussion about the different architectures and we do not see any reason for RNN to behave differently from the architectures we tested.

-Relating specifically to the reviewer’s question whether a latent space is required, we want to emphasize that while we see significant improvement in prediction performance when using latent spaces or informative baselines for the splicing task, EIG is not limited by the use of an autoencoder. Users can choose to work with linear or neighbor based paths in the original feature space while making use of the group specific baselines. To illustrate this, we show significant features found by a CNN with or without a convolutional variational autoencoder (CVAE) on the problem of handwritten digit recognition (Figure 2b: CNN without CVAE, using median baseline with linear path, Figure 2c: CNN with CVAE, using median baseline with latent-linear path). Only users that want to make use of the latent paths or encoded-zero baseline need to train an autoencoder. We also emphasize that the need for latent spaces can be problem specific, with linear paths working well for certain problems. For example for handwritten digit recognition, users can stick to linear paths. However in cases when linear paths with naive zero baseline fails, e.g. splicing code, training an autoencoder is not a significant overhead to find relevant biological signal.

4. It's not clear that an autoencoder is required to apply the method - one can view the hidden neurons of a network trained using supervised learning as an embedding instead of having to perform an additional step of training an autoencoder. Please comment on that.

- The reviewer raised a good point that requires clarification. In general, as noted above for using EIG users can employ the group specific baselines and the significance framework regardless of the availability of an autoencoder. In cases where the user wants to compute paths in the latent space, working with an embedding of the supervised neural network is not sufficient since these paths need to be decoded to the original feature space to produce attributions in the original feature space. An autoencoder provides such a decoder, making the autoencoder necessary for working with latent paths.

5. The authors use the number of significant features that a method discovers as an indication of its quality. However, in the context of feature selection, the goal is typically to find a small set of features that provide accurate performance. Admittedly, the finding that their LIG method finds significant features where the original IG method does not, is an indication of better performance, it's difficult to make a case that finding say 500 is better than 200, and the authors admit that.

-We agree with the reviewer that finding more features is not necessarily an indication of the quality of a method. As pointed out by the reviewer, EIG finds significant features where the original IG method fails, which is an indication of better performance but we need additional criteria for comparing performance of different interpretation methods introduced in the paper.

Therefore, we used two evaluation metrics: enrichment of previously reported biological features in the set of identified features and the predictive performance using the chosen significant features.

Figure 3e: shows the enrichment for group agnostic encoded-zero with O-L-IG and H-N-IG paths, group specific median baseline with O-L-IG and H-L-IG paths and DeepSHAP. Performance of other methods is shown in the Supplementary figure S7. Higher enrichment indicates that the interpretation method is finding these features at higher frequency than their competition. While far from perfect (e.g. cannot give accurate measures of FP and FN) this evaluation is one proxy for the quality of features found by an interpretation method. This evaluation was present in the paper before this revision for the best four EIG paths and DeepSHAP. However, to address the concern raised by the reviewer we have added the performance of other methods in the supplementary figure S7. Significant features found with Median O-L-IG and H-L-IG showed the highest enrichment of these curated features.

Furthermore, to evaluate the predictive performance, we perform two types of tests. First, we compare the performance of increasing sets of significant features (or meta-features) with randomly selected features (or meta-features) for handwritten digit recognition and splicing in brain prediction tasks. Figure 2e shows this evaluation on the binary classification task for digits (5 and 3). Since the EIG methods aim to find signal relevant for distinguishing the baseline digit (3) from the sample digit (5), we chose these two digits for the binary classification task. For splicing task, figure 3f shows this comparison for the task of differential inclusion of splicing events in the brain. For both tasks, the deep learning model performs better when using the significant features/meta-features compared to random feature/meta-feature selection. Median H-L-IG was used to get the list of significant features in both cases.

Using Figure 2e and 3f, we can answer the question: How many features are needed for best predictive performance? For the handwritten digit recognition, the graph plateaus around 30-48 significant features (figure 2e) and for splicing prediction task, the saturation is 70-100 significant meta-features.

More generally, we find that combining the graphs of empirical p-values distribution (see below) with the graph for prediction accuracy as a function of #informative features can give insights into the domain at hand: How many features are informative? Is the solution sparse (small number of features)? Are the features redundant in their predictive capacity? We added a new subsection describing this to the main text.

Then we evaluate the predictive performance of all methods (paths: O-L-IG, H-L-IG, O-N-IG and H-N-IG and baselines: zero, encoded-zero, median, k-means, close, random) on the same splicing task of differential inclusion of splicing events in brain. We find that all flavors of EIG that find more that 70-100 meta-features, show similar predictive performance (Figure 3g). Exceptions are simple gradients, zero baseline with O-L-IG and H-N-IG paths.

Together, these evaluations show that most EIG flavors perform similarly in terms of predictive performance on the task of splicing prediction, but different methods have different enrichment of known biological features with median O-L-IG and H-L-IG showing highest enrichment of these features.

In summary, we have added several analysis of different performance criteria, with matching figures, to the revised manuscript. To be clear, we do not think the above is a complete and definite, quantitative, answer regarding the best approach which could quite likely be domain specific. For example, the nature of the features (binary, discrete, real), their dependency and correlation structure, the sparsity of the relevant feature set - all of these are factors that can affect relative performance for a specific domain of interest. We specifically added a paragraph about the need for cross domain insights into how such methods work and why to the discussion. Nonetheless, we believe we offer both methods improvements and some insights into what methods work better for the domain we focus on (splicing codes).

6. Summing up the contributions of features that are part of a meta-feature is not a good idea in my opinion - it provides an inordinately large weight for such features, and make it such that they are more likely to be selected compared to others. Taking the maximum of a cluster of features would be more appropriate.

The reviewer raises an interesting point. While we agree that summing up attributions for meta-features might inflate weights for large meta-feature, taking the maximum of the cluster can have the opposite problem, where large sets of highly correlated/redundant features will be unnecessarily penalized as the attributions are distributed amongst more members, making them less likely to be selected compared to some small meta-feature when they might actually be more informative. Regardless, we note that for each meta feature we assess the distribution of the weights compared to a null such that the size/identity of the meta feature (and associated sum of its features weights) is calibrated with respect to that when assessing significance (Supplementary Figure S8).

Minor comments

1. In some areas the Results section is not clear - mentioning methods or terms that have not been discussed (downside of a Results-first paper organization). For example, "our autoencoder" is mentioned before the reader has any notion how those were used. Similarly, at the paragraph at the top of page 3, second column, more background is required to understand the discussion.

We thank the reviewer for noticing this and have added the requisite background in both places.

2. It was not clear if feature selection was performed on the training set only. Please clarify that.

We are not exactly sure about the context of this question, we will try to answer it to the best of our understanding. We train our splicing and digit classification models on the training set and evaluate performance on the test set. We remove features with zero variance from the training set for the splicing task (number of features go down from 1357 to 1260 after this selection) but don’t do any further feature selection for the initial model training. 1357 splicing features that we use are derived from older splicing codes publications (Barash et al 2010, Jha at el 2017). For handwritten digits, we run the initial models with all 784 pixel features.

3. The nearest neighbor path method doesn't seem to perform as well and is more computationally expensive. Consider removing it to improve the readability of the manuscript (could be mentioned that such a method was considered, but did not perform at the same level as the other proposed variations). The results section presents many variants that make it challenging to keep track of all of them.

We agree with the reviewer removing the nearest neighbour path method would simplify the paper, this is a valid concern. However, after much deliberation we decided to retain it for the following reasons: While the nearest neighbors path is computationally expensive and not the best performer for the splicing task, it does seem to perform well in some cases when other methods fail (Figure 3a: zero baseline O-N-IG) and shows second best enrichment compared to other paths with the encoded-zero baseline (Figure 3e. Encoded-zero H-N-IG). Additionally, we suspect the nearest neighbors paths could be especially useful for interpretation of single cell RNA-Seq data. Given the above we view it as a potential impactful contribution worth including in the main text. However, we did aim to address the reviewer’s concern by improving readability and notation (see also above).

4. Set_R and Set_G are not defined in the text and are not really needed outside of Algorithm 1. Algorithm 1 itself needs to be more clear (e.g. what are G and R).

We regret this omission and have added further explanations about Set_R and Set_G to the text. To clarify: The input G contains attributions for all features/meta-features for a set of events of interest (e.g. differentially included exons in brain) and R contains attributions for all features/meta-features for a random set of events. Set_G is a subset of G with absolute attribution for one feature/meta-feature for all examples from a class. Set_R is a subset of R with absolute attribution for one feature/meta-feature for a random subset of examples from all classes. For each feature/meta-feature, we perform a one-sided t-test for the positive tail of the distribution on Set_G and Set_R.

5. k-means and median baseline - in which space are these computed?

These baselines are computed in the original feature space. We have added this information to the text.

6. You mention that you analyzed "a significant meta-feature identified through this procedure" - exactly which procedure are you referring to? LIG has several flavors. Were all of them able to detect it? What made you choose that particular meta-feature?

Our analysis for enrichment of known biological features showed highest enrichment for median baseline followed by encoded-zero baseline. Therefore, we ran the differential inclusion/exclusion in all six tissues (hippocampus, heart, liver, lung, spleen, thymus) analysis using encoded-zero and median baselines for all four paths (O-L-IG, O-N-IG, H-L-IG and H-N-IG). We found enrichment of A1CF motif feature in the liver runs for the following paths: encoded-zero H-N-IG, median O-L-IG, O-N-IG, H-L-IG and H-N-IG. We selected A1CF after doing a literature search over the top informative features for the liver set. We added a more detailed description to clarify these points in the main text.

Grammar/notation/references

- Ref 9 was published in ICML.

- Check capitalization of references (e.g. ref 15)

- "First the distances are computed on the L" --> are computed in L or are computed in the latent space.

- linear paths in original and latent space --> the original and latent spaces

We thank the reviewer for pointing out these errors. We have updated these in the text.

Reviewer #2: (Remarks to the authors) The manuscript by Jha et al. describes strategies to evaluate feature importance for deep learning models using integrated gradients (IG). This work addressed several limitations of the original IG method: 1) selection of reference, as a zero is frequently not meaningful for genomic data; 2) the path of integration from reference to the sample; 3) a significance test to evaluate the importance of features related to the classification task instead of a specific sample. These improvements are very useful for effective interpretation of deep learning models in many potential applications. The authors demonstrated effectiveness of the proposed improvements in digit application and tissue-specific alternative splicing prediction. Interestingly, the authors found A1CF as a regulator of liver specific splicing. Overall, this manuscript provides very useful insights into an important topic. However, the manuscript can be improved in many ways for rigor and clarity.

Comments

1. The authors use splicing code as a major application, and mentioned several RBPs such as Rbfox and Nova. It will be helpful to provide some introduction of these RBPs and other features used for prediction.

We completely agree and thank the reviewer for this suggestion. We have added text describing regulatory features and specifically these RBPs to make the domain more understandable.

2. P3, The authors clustered original features into meta-features. How many meta-features after grouping correlated features?

We cluster 1357 features into 781 meta-features. We have added this information to the text.

3. P3, in the section, "IG with nonlinear paths identify significant features missed using linear paths or simple gradients". The authors should explicitly state that the results are from zero baseline in the original feature space.

We have added this to the text.

4. Baseline group and group of interest should be defined more clearly.

We now include the following text to make the term more clear: “Specifically, for the results described below, we use a group of cassette exons that are differentially included in hippocampus as the set of interest. These alternative exons should be enriched in features informative for differential inclusion in hippocampus. We select constitutively spliced exons that are always included in all tissues as the baseline class. Constitutive exons are chosen as the baseline since these events differ from alternative events in both core splicing features and brain specific splicing features.”

5. P4: calibration of p-values using random group of events. It will also be helpful to plot the distribution of p-values in the real dataset.

We want to particularly thank the reviewer for this comment which we found to be very helpful. First we added the plot showing *p*-value distribution for both the digit recognition task (Supp Fig S9) and the splicing prediction task (Supp Fig S10). But more importantly, we used these plots, combined with the the plots showing accuracy as a function of # informative features (Main Fig2e, Fig3f) to gain insights about the informative features in the domain: Do they represent a sparse solution? Are they redundant in their predictive accuracy? Do the rest of the features follow the null? We added a section about this in the main text and want to thank the reviewer for making the above suggestion.

6. P4, enrichment of known biological features - details are missing completely. Which features are "known biological features"?

We apologize for not including a description of how this set was derived. We added the following to the Methods section:

Defining the set of known biological features: As described in the main text, the set of splicing code features used in this work has been previously curated from the literature and is therefore highly enriched in informative splicing regulatory features in general, and for regulation of splicing in muscle and brain in particular (Barash et al 2010, Barash et al 2013). To define a high-quality set of known biological meta-features from these for our specific task of interest we therefore ran a hypergeometric test to compute p-values for enrichment or depletion of a feature in the differentially included splicing events in the brain compared to a negative set of constitutive splicing events. To get a high-quality feature set we used a highly conservative threshold of -log(p) > 20 which resulted in

several hundred features from those initially selected for the splicing code. Finally, we combined correlated features as meta-features and assigned the minimum p-value assigned to a member of the meta-feature group as the p-value for that meta-feature.

7. P4, "Identified significant features improve splicing prediction" - not clear what does that mean from the comparison of 70 meta features vs 142 original features. It will be more informative by comparison of significant features vs. a set of randomly selected features or a set of features selected by a baseline method of equal size.

We thank the reviewer for this good suggestion. We have added the experiment that compares performance of significant features/meta-features with random features/meta-features for handwritten digit recognition (Figure 2e) and the splicing task (Figure 3f). Both these comparisons show that features/meta-features selected by EIG lead to better predictive power than randomly selected features/meta-features.

8. Fig. 3a, poor visibility of the images with background.

We apologize for the poor visibility of the original images and have updated the figures with a gray background instead of black to improve visibility of Figure 1b, 1c and 2a. We have added the new figures (Figure 2b and 2c) with the gray background as well.

9. Fig. 4 cd, the authors interpreted some of the peaks as biological important. Overall, the RNA map in panel c is quite noisy, and it is unclear if peaks are real or due to random fluctuations. It will be helpful to distinguish liver-specific exons by liver-specific inclusion/skipping, and similarly A1CF dependent exons by activation/repression, and then analyze motif enrichment in exon, upstream/downstream introns separately.

We thank the reviewer for pointing this out and suggesting interesting follow up analysis that we agree clarify our results. To address these concerns and expand on the suggested analysis we have added a new supplemental figure (now Figure S10) and added an additional panel to now Figure S11. To address the issue of peaks looking noisy, in all motif maps we have added gray-scale boxes to indicate regions of significant differences in motif frequencies between the various exon sets to more clearly and directly point out which peaks are meaningfully different. As suggested we have stratified our exon sets ino A1CF enhanced and repressed exons and compare those results to liver-specific inclusion and exclusion exons in the motif map analysis (Figure S10) as well as for the PAR-CLIP binding analysis (Figure S11c).

10. Fig. S1: label. It is weird to have "genomic features" and "tissue" for a digit classification task. The labels should be corrected.

11. Fig. S2: 2b, encoder layer 1 -> decode layer 1

We thank the reviewer for pointing out these errors. We have updated them in the text.

**2^nd^ Round**

**Reviewer 1**

The authors have addressed my original concerns regarding the manuscript.

Thanks for the clarification on which aspects of the method require an auto-encoder.

The authors may want to consider making the abstract more explicit on the contribution of their work, which provides significant improvements of the original IG, making it more usable for genomics and other application areas.

Typos and minor comments:

In summary, we found that monitoring that p-value distribution for EIG feature attribution, --> the p-value distribution

**Reviewer 2**

I have no further comments on the manuscript.
